# Interfacial Interactions between Neural Tracing Lectin–Gold Nanoparticle Conjugate and Cell Membrane Glycoproteins

**DOI:** 10.1021/acs.langmuir.4c05034

**Published:** 2025-04-16

**Authors:** Joel Yong, Dan Wang, Lachlan Kwok, Sk Al Zaheri Mahmud, Karen Hakobyan, Megan S. Lord, Guangzhao Mao

**Affiliations:** † School of Chemical Engineering, University of New South Wales, Sydney 2052, Australia; ‡ Graduate School of Biomedical Engineering, University of New South Wales, Sydney 2052, Australia; § School of Engineering, Institute for Materials and Processes, The University of Edinburgh, Robert Stevenson Road, Edinburgh, EH9 3FB, U.K.

## Abstract

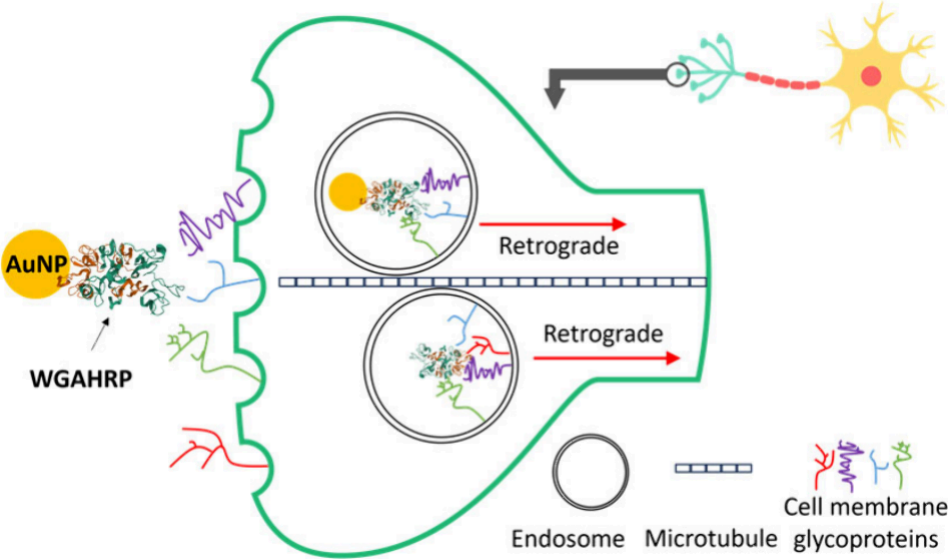

The use of neural tracers as targeting molecules for drug delivery has been previously established as a novel and efficient method of neural drug delivery. The wheat germ agglutinin–horseradish peroxidase conjugate (WGAHRP) is a common neural tracer, which has been used extensively to decipher neural pathways in vertebrates. It has been widely reported to bind to cell surfaces and be transported in a retrograde fashion (from synapses toward the cell body) via dynein motors along microtubules within axons and transynaptically between neurons. Here we report on the differential binding between WGAHRP and gold-conjugated WGAHRP (AuNP-WGAHRP) to the glycoprotein profiles extracted from two neuronal cell lines and one skeletal muscle cell line, as well as the binding kinetics to heparin. From proteomic analysis of the extracted glycoproteins, we suggest the identity of cell surface glycoproteins involved in the retrograde transport of WGAHRP. This study illuminates the interfacial and molecular interactions of protein–gold conjugates with native ligands and opens the door for the identification of new targets for neural tracing and nervous system-related drug delivery.

## Introduction

Drug delivery to the central nervous system (CNS) is notoriously difficult because of the inherent structural barriers which tightly regulate the microenvironment of the brain and spinal cord. New drug delivery systems, which can selectively transport therapeutic molecules to regions of interest, in an efficient and effective manner, could revolutionize the treatment of CNS disorders. In the past, our group has utilized WGAHRP as a targeting mechanism to deliver therapies to the brain in a spinal cord injury model, in conjunction with gold nanoparticles (AuNP) as a drug carrier.^1−3^ This work involved the use of an AuNP-WGAHRP-drug conjugate to demonstrate the highly efficient delivery of adenosine receptor antagonistic drugs, 1,3-dimethylxanthine (theophylline) and 1,3-dipropyl-8-cyclopentylxanthine (DPCPX), via the phrenic nerve in a rat spinal cord hemisection injury model, completely bypassing the blood-brain barrier and blood-spinal cord barrier. We have elucidated the main cellular uptake pathways (clathrin-mediated) and retrograde transport kinetics of the nanoconjugate (1.4 ± 0.5 μm/s) using dorsal root ganglion (DRG) cells.^4^ However, considering the wide range of membrane proteins WGAHRP can bind to (via sialic acid or *N*-acetyl glucosamine (GlcNAc)),^5,6^ the molecular binding partners of WGAHRP-mediated retrograde transport remain unknown. Our previous work^4^ demonstrated no alteration to neuronal cell uptake or retrograde axonal transport after WGAHRP conjugation to gold nanoparticles. However, a facile method to directly determine the ligand binding affinity to membrane proteins will speed up the development process for this new class of nanomaterials. In the interest of developing drug delivery platforms via neural tracers, these two aspects, ligand binding affinity and retrograde transport-coding binding partners, would be informative for the optimization of these platforms through more efficient targeting and chemistry.

Colloidal AuNPs are a popular choice for nanoparticle-mediated drug delivery, having been studied extensively in recent years.^7^ Their size can be easily controlled and their affinity with thiol groups allows for simple conjugation chemistry with many molecules. Additionally, they are easily imaged via TEM and two-photon microscopy. In the case of drug delivery, the surface area of AuNPs allows for delivery of a higher dose of drug as compared to a direct conjugation between WGA and a drug, which is a recent development and has yet to be tested for drug delivery. The AuNP size used in this paper was previously optimized at 4 nm for trans-synaptic transport,^1^ according to the size limitation of synaptic clefts (20 nm).^8^

WGA is a lectin purified from *Triticum vulgaris* that binds to GlcNAc and *N*-acetyl neuraminic acid (sialic acid/NeuC) sugar residues, which are found on sugar-decorated (glycosylated) proteins throughout the animal, fungi and bacteria kingdoms.^9,10^ The use of WGAHRP as a neural tracer in both anterograde and retrograde directions has been widely reported among neuroscientists tracing neural pathways since the late 1970s,^11^ being able to cross neuromuscular^12^ and synaptic junctions.^13^ Fluorescent label conjugated formulations are used commonly as a cell membrane stain and is able to survive fixation.^14^ Horseradish peroxidase (HRP, from *Amoracia rusticana*) is an enzyme with an iron-binding heme catalytic center commonly used as a conjugate for histological staining, chemiluminescence, and colorimetric detection. The conjugation of these two proteins has been reported to produce greater retrograde axonal transport capabilities than HRP alone (which is not transported trans-synaptically),^11^ and higher sensitivity than WGA alone.^13^ Other popular neural tracers (covered in our recent review^15^) include viral proteins, bacterial toxins and live attenuated viruses. Many of these exhibit differential staining of neuronal populations compared to WGAHRP, owing to their differential binding ligands and subsequent trafficking. If properly characterized, these differential staining patterns can potentially provide mechanisms for targeting specific neuronal populations.

WGA exists as three major isoforms (isolectins, WGA1, WGA2, and WGA3) and occurs naturally as a 36 kDa dimer, containing 8 binding sites for GlcNAc^5^ and 2 binding sites for sialic acid sugars.^6^ Each monomer consists of 4 domains, named hevein A-D, each consisting of 43 amino acids. The high cysteine and glycine content of these domains provides a highly stable dimer. GlcNAc and sialic acid residues are present as part of oligosaccharide structures attached as post-translational protein modifications to specific amino acids in the Golgi apparatus. Proteins decorated with oligosaccharide structures are known as glycoproteins and are broadly classed as O-linked or N-linked (linked to serine/threonine or asparagine, respectively). Interestingly, HRP has 8 N-linked sites containing GlcNAc (as part of several heterogeneous glycan structures), which constitute ∼18–22% of the molecular weight.^16,17^ Initiation of glycosylation (the post-translational process of modifying proteins with sugars) begins in the endoplasmic reticulum, during which specific saccharide structures are attached and then trimmed during protein folding, after which they are transported to the Golgi apparatus for maturation by a myriad of enzymes into linear or branched oligosaccharides with various compositions.^18^ There are 10 different monosaccharides required to build over 7000 identified glycan structures: glucose, galactose, sialic acid, mannose, fucose, GlcNAc, *N*-acetyl galactosamine (GalNAc), uronic acid, xylose, and ribitol.^18^ These structures can vary in size from a few thousand, to hundreds of thousands (heparin, heparan sulfate, chondroitin sulfate glycosaminoglycans), to even millions of daltons (hyaluronan). Thus, the glycoprotein coat of cells (glycocalyx) forms a heterogeneous coat of sugar chains extending into the extracellular space, whether that be synaptic, extracellular matrix, luminal, or otherwise. The thickness of this coat is dependent on the cell type and location, ranging from a thickness of 2–5 μm in endothelial cells of blood vessels, with a thicker glycocalyx in larger blood vessels.^19^ Logically it follows that the glycocalyx composition is key to WGA-mediated retrograde transport. However, since many glycoproteins contain sialic acid and/or GlcNAc, it is unknown which glycan structures or which glycoproteins provide the correct signals’ coding for retrograde transport in neurons. For AuNP-WGAHRP-drug conjugates, the envisioned delivery and subsequent transport route is as follows: intramuscular injection, crossing of neuromuscular junctions into peripheral neurons, followed by retrograde and trans-synaptic transport within and between neurons, toward the central nervous system. To model the path of a retrograde drug delivery route via intramuscular delivery, we chose three cell lines, a muscle cell line (C2C12), a cell line derived from motor neurons from the spinal cord (NSC-34) and a cell line derived from neurons from the brain (NE-4C). The glycoproteins from these cell lines provide a good sample from which to investigate AuNP-WGAHRP binding ability and determination of important glycoproteins for WGAHRP-mediated retrograde transport.

In this work, AuNP–WGAHRP conjugates and their constituent parts were characterized, and their binding to native ligands compared against WGAHRP via several methods. Specific binding of AuNP–WGAHRP to native ligands was confirmed; however, a direct comparison with WGAHRP was not possible. This comparison by standard biochemical methods was made difficult by two special properties exhibited by AuNPs—acceleration of peroxidase activity via direct electron charge transfer and fluorophore quenching via nanometal surface energy transfer. These properties may be of use in biosensor applications. By manual curation of glycoproteins identified through mass spectrometry, a nonexhaustive list of potential candidates was generated, which may represent some of the binding partners that allow retrograde axonal transport of WGA.

## Experimental Section

### Materials

Chemicals were purchased from Sigma-Aldrich and Thermofisher Scientific. For a full list, see Supporting Information.

### Cell Culture

Neuroblastoma x spinal cord-34 (NSC-34, mouse), Neuroepithelial-4C (NE-4C, mouse), and C2C12 mouse skeletal muscle cells were cultured in T25 flasks in DMEM supplemented with 10% (v/v) FBS and 1% (v/v) penicillin-streptomycin (P/S). Cell media was changed once a week, and cells were passaged using TrypLE cell dissociation reagent. Images were captured on a microscope (IX73, Olympus) with a 10× lens via a charge-coupled device (CCD) camera (XM10, Olympus) with Olympus Cellsens v2.3 software.

For differentiation of NSC-34 cells, culture vessels were coated with 30 μg/mL laminin (isolated from Engelbreth–Holm–Swarm sarcoma) for 2 h at 37 °C. Subsequently, NSC-34 cells were harvested using 20 mM EDTA in PBS at pH 7.2, washed twice with 10 mL of PBS. Laminin was removed from flask, and NSC-34 cells were quickly seeded in differentiation media consisting of Neurobasal Plus media with 1% CultureOne supplement. Neuron-like cells with long axonal and dendritic processes were seen within 1 day. Cells were allowed to differentiate for at least 7 days.

For differentiation of NE-4C cells, cells were plated at a high density (∼80–120 × 10^5^ cells/cm^2^) in growth media and allowed to adhere for 4 h in the incubator. Following this, media was changed to Neurobasal Plus media with 1% B27 supplement and 10 mM IGF-1. Three days later, massive cell death occurred, leaving differentiated cells attached to the flask surface. Media was replaced every 3 days for another 10 days.

For differentiation of C2C12 cells, cells were allowed to grow to confluence, before changing to differentiation media consisting of Neurobasal Plus media with 1% N2 supplement and 10 nM IGF-1. Cells were allowed to differentiate for at least 14 days.

### Expression of Differentiation Markers by Confocal Microscopy

Cells were differentiated on glass slides as described above. Cells were fixed with 4% PFA in PBS for 15 min at room temperature, washed twice with PBS, permeabilized with 0.1% Triton-X in PBS, blocked with 1% BSA in PBS overnight at 4 °C and probed with antibodies for 2 h at room temperature (see Supporting Information for details). Cells were then washed twice with PBS and probed with goat antirabbit IgG AlexaFluor 633 secondary antibodies (4 μg/mL), WGAHRP–ATTO488 at 5 μg/mL and Hoechst 33342 (10 μg/mL) for 1h at room temperature. Following this, cells were washed again twice with PBS-T and twice with PBS, and coverslipped with Prolong Diamond antifade mounting solution. The mounting solution was allowed to cure for at least 24 h at room temperature before imaging on a confocal microscope (LSM 800, Zeiss).

### Gold Nanoparticle Synthesis and Conjugation

Gold nanoparticles were synthesized as reported previously.^1,4^ Glassware was acid washed (aqua regia, 3:1 ratio of 32% HCl: 70% nitric acid) for 2 h and rinsed copiously with Milli-Q water. Sodium citrate tribasic dihydrate (25 mg) was dissolved in Milli-Q water (248 mL). Gold(III) chloride (25 mg) was dissolved in water (1 mL) and added to the citrate solution with very strong stirring. Nanoparticles were precipitated by the addition of sodium borohydride (20 mg) dissolved in water (1 mL). The solution turned from a light yellow to a wine red color within 2 s. After 1 h of stirring, the pH of the solution was increased to 11 by adding 0.1 M NaOH dropwise. The resulting solution was filtered using a 0.22 μm syringe filter. The filtered nanoparticle solution was then reacted with mercaptosuccinic acid (MSA, 25 mg dissolved in 0.5 mL water) overnight at room temperature to replace the citrate with succinate. The resulting MSA-capped AuNP solution (designated AuNP–MSA from hereon) was washed with Milli-Q water using 10 kDa molecular weight cutoff centrifugal (MWCO) filters (Amicon UFC9010), at 2600 RCF for 8 min *per* wash. Particles were washed 3 times. Subsequently, the AuNP–MSA solution was brought back to the original synthesis volume (250 mL). From this, a portion (45 mL) was taken for conjugation with WGAHRP. Under strong stirring, EDC (0.9 mg in 0.5 mL water) was added to the solution and allowed to react for 5–10 min, before NHS (0.45 mg in 0.5 mL water) and WGAHRP (0.66 mg at 1 mg/mL) were added. The solution was allowed to react for a further 1 h at room temperature before washing and concentration as above. The AuNP-MSA-WGAHRP conjugate, designated AuNP–WGAHRP hereon, was stored and used as a 2× concentration (i.e., 0.2 mg/mL Au synthesis basis) unless otherwise stated.

### Multiangle Dynamic Light Scattering (MADLS) and Zeta Potential

Hydrodynamic radius and zeta potential were measured using a Zetasizer Ultra Red (Malvern Instruments). One mL of filtered nanoparticle solution (0.22 μm) was placed in a fresh plastic cuvette (ZEN0040), and the hydrodynamic radius was measured in triplicate in multiangle mode (forward scatter, 13°; side scatter, 90°; back scatter, 173°) with the refractive index set to 1.45 and the absorbance set to 0.001. Hydrodynamic radius was calculated from the autocorrelation function using a nonlinear least-squares fit automatically by the software. Zeta potential measurements were performed in triplicate in a disposable plastic (DTS1070) zeta cell.

### Nanoparticle Tracking Analysis (NTA)

NTA was conducted using a Nanosight NS300 (Malvern Instruments) featuring a 658 nm laser, 20× objective, scientific CMOS camera and NTA software version 3.4, build 3.44. AuNP-MSA and AuNP-WGAHRP were diluted to a 0.5× concentration using Milli-Q water and filtered using a 0.22 μm syringe filter. Samples were injected using a syringe pump (Cat# 98-4730, Harvard Apparatus) through a bubble trap (LVF-KBT-S, Darwin Microfluidics) and into the low volume flow cell (NTA0065 + NTA0066, Malvern Instruments). Once the sample was visible on screen, the camera was focused and video data collected for 60 s. This was repeated five times for each sample, advancing the sample between repeats. For particle tracking, the detection threshold on the software was set to 3, and the maximum jump distance and minimum track segment length were set to auto. Particle size distribution was automatically calculated by the software using the Stokes–Einstein equation and refined using finite track length adjustment (FTLA). The flow cell was purged with filtered Milli-Q water several times between samples.

### Transmission Electron Microscopy (TEM)

Formvar coated copper grids (200 mesh, Cat# 01810, Ted Pella, Inc.) were treated with oxygen plasma using a Pelco easiGlow Glow discharge cleaning system (Ted Pella, Inc.). Nanoparticle solution was diluted 10 times in Milli-Q water, and 7 μL was dropped onto the grid. After waiting for 5 min, the excess liquid was wicked off using filter paper. Grids were allowed to dry at room temperature in a grid holder before imaging in a transmission electron microscope (Tecnai G2 20, FEI) operating at 200 kV with a CCD camera exposure of 0.5 s. Particle diameter was measured manually using Fiji (ImageJ) and analyzed using GraphPad Prism v10.0.3.

### UV–vis Spectroscopy

UV–vis (Nanodrop One, Thermo-Fisher) was conducted using 2 μL of samples, which were placed on the pedestal after backgrounding. For gold nanoparticles, absorbance was measured over the range of 200–800 nm and smoothed using a second-order smoothing function with 20 neighbors. For glycoprotein extractions, protein concentration was estimated using absorbance at 280 nm, assuming 1 AU = 1 mg/mL.

### Elemental Analysis

WGAHRP concentration in the AuNP–WGAHRP sample was estimated by nitrogen elemental analysis. Nitrogen concentration was determined via the Dumas combustion method (thermo-catalytic high temperature oxidation) in a Multi N/C (3100, Analytik Jena). Briefly, 500 μL of aqueous sample was combusted at 800 °C in a grade 5.0 oxygen atmosphere in the presence of a platinum catalyst and detected by chemiluminescence. The nitrogen concentration was then converted to a WGAHRP concentration by determining the nitrogen content of WGAHRP via the amino acid sequence of the mature peptide forms of both WGA and HRP (UniProt IDs P10969 and P00433 respectively). See Supporting Information for full details.

The total concentration of Au was carried out using a multiquadrupole-based inductively coupled plasma mass spectrometer (NexION 5000, PerkinElmer, USA). The instrument operating conditions were set as follows: wavelength 213 nm, repetition frequency (RF) 10 Hz, laser energy density 4.8 J/cm^2^ (at 30%), spot size 110 μm, depth 1.5 μm and a scan speed 20 μm/s. ICP-MS was performed at a RF power of 1200–1500 W, plasma gas flow rate of 15–17 L/min, auxiliary gas flow rate of 1–2 L/min and nebulizer gas flow rate of 0.85–1.2 L/min. The working solution was aqua regia and the operating software was Syngistix for ICP-MS. The optimization of pulse and analogue stages of the detector were done by using Au 197 masses of gold at standard mode where the internal standard was Iridium-193. The instrument was calibrated with freshly prepared series of standards from a stock standard solution which includes the required element (at least 4 points including blank) before starting analysis of the samples. Total concentration of elemental gold was used to calculate AuNP concentration. See Supporting Information for more details.

### Glycoprotein Extraction

Cells were lysed in T25 cell culture plates using 1 mL RIPA buffer (ThermoFisher, Cat. 89900) with EDTA free protease inhibitor (ThermoFisher, Cat. A32965) that had been sterilized using a 0.22 μm syringe filter. Lysates were spun at 17000*g* for 20 min, before the supernatants were extracted, buffer exchanged with PBS and concentrated using 10 kDa MWCO centrifugal filters (14000*g* for 15 min per spin) (Sigma-Aldrich, UFC5010). Protein concentration was determined using UV–vis. WGA-binding glycoproteins were extracted using a commercial kit (Thermo-Fisher, Cat. 89805) according to manufacturer instructions. Crude lysate protein (1.5 mg) was mixed with 5× binding buffer at a 4:1 protein to binding buffer ratio. This mix was incubated with 50% WGA-resin slurry (200 μL) for 30 min and washed 3 times with a wash buffer. Washed resin was incubated with elution buffer (200 μL) on a mixer for 10 min, and then eluted at 1000*g* for 1 min. This was repeated one more time to collect two fractions, which were pooled. Pooled eluate was buffer exchanged with PBS, and concentration was determined using UV–vis.

### Sodium Dodecyl Sulfate Polyacrylamide Gel Electrophoresis (SDS-PAGE)

Sample preparation: 170 μL of AuNP-WGAHRP (33 μg/mL WGAHRP basis, equating to roughly 5 μg of WGAHRP) was concentrated to ∼30 μL using a 10 kDa MWCO centrifugal filter. 15 μL of this concentrate was used in SDS-PAGE (equating to 2.5 μg WGAHRP). This was repeated for AuNP-MSA as a control. To check conjugation efficiency, we opted to precipitate AuNP-WGAHRP and test the supernatant. AuNP-WGAHRP was precipitated by mixing 170 μL with 470 μL of a salt solution (3.4 M MgCl_2_.6H_2_0 and 9.2 M CaCl_2_.2H_2_0), heating at 37 °C for 15 min and then centrifuging at 17900*g* for 20 min. This produced a black pellet. The supernatant was extracted, buffer exchanged with Milli-Q water and finally concentrated to 30 μL. Fifteen μL of this was used in the SDS-PAGE. 5 μg of protein samples were used in SDS-PAGE.

Samples were mixed with 4× Laemlli buffer (Biorad) at a ratio of 3 parts sample to 1 part Laemlli buffer (v/v). For reduced samples, samples were reacted with tris(2-carboxyethyl)phosphine (TCEP) in water at a final concentration of 50 mM for 30 min before neutralizing with NaOH, after which they were mixed with 4× Laemlli buffer.

Samples were loaded into Biorad Mini-PROTEAN TGX precast gels (Biorad Cat. 4569035) and electrophoresed at 200 V for 30 min in Tris-glycine running buffer consisting of Tris (25 mM), glycine (0.192 M) and SDS (1% w/v). The Biorad Precision Plus molecular weight ladder (5 μL per lane) was used as molecular weight standard. Following electrophoresis, gels were washed on a rocker three times with Milli-Q water for 5 min each, before staining in SimplySafe solution for 1 h at room temperature on a rocker. The gel was then destained by washing the gel in Milli-Q water three times, 20 min each. The gel was imaged (Chemidoc MP, Biorad) using the Coomassie Blue Gel filter (715 nm/30) for Coomassie-stained proteins or SYPRO Ruby filter for AuNP samples.

### Western Blot

First, 0.2 μm polyvinylidene fluoride (PVDF) membranes were activated for 5 min in methanol. Transfer was conducted in Towbin buffer (25 mM Tris, 192 mM glycine, 20% v/v methanol, pH 8.3) at 200 mA for 2 h using a Mini Trans-Blot cell (BioRad) cooled with ice water. Following transfer, blots were imaged using BioRad Clarity Western ECL substrate and imaged (Chemidoc MP, Biorad).

### Enzyme-Linked Lectin Assay (ELLA) and Fluorescence-Linked Immunoglobulin Assay (FLISA)

Extracted glycoproteins were immobilized on high-binding 96-well plates (Greiner 655081) (50 μL *per* well) at a concentration of 4 μg/mL for 1 h at 37 °C on a shaker (Benchmark Multi-Therm). Wells were washed twice with PBS before blocking with 0.5 w/v% PVA for 1 h. Wells coated with PVA (0.5% w/v) was used as a negative control. Wells were washed twice with Tween-20 in PBS (PBS-T, 1% v/v), and incubated with WGAHRP (1 μg/mL) or AuNP–WGAHRP (1 μg/mL WGA-HRP), diluted in PBS containing 1 mM CaCl_2_ (1 mM) and MgCl_2_ (1 mM) for 2 h at room temperature. Wells were washed again, twice with PBS-T and twice with PBS before adding 2,2′-azinobis [3-ethylbenzothiazoline-6-sulfonic acid]-diammonium salt (ABTS) solution, consisting of 10 mg ABTS in 10 mL of phosphate-citrate buffer (24.3 mM citric acid, 51.4 mM dibasic sodium phosphate) with 0.03 v/v% H_2_O_2._ Color development proceeded for 15 min before reading the absorbance at 405 nm in a plate reader (Clariostar, BMG). The absorbance was saturated within 20 min. For the fluorescence-linked immunoglobulin assay, following absorbance reading, wells were washed twice with PBS-T and probed with lectin antibodies at a concentration of 5 μg/mL in 1% BSA/PBS, for 2 h at room temperature on a shaker. Wells were washed again twice with PBS-T and probed with goat anti-rabbit AlexaFluor 633 antibodies at 10 μg/mL in 1% BSA/PBS for 1 h at room temperature on a shaker. Wells were washed twice with PBS-T and twice with PBS. Fluorescence was read on the plate reader (Excitation 610/30 nm, emission 675/50 nm) with manual gain.

### Microscale Thermometry (MST)

The binding between FITC-labeled heparin (60 nM, FITC-heparin, 27 kDa, Creative PEGWorks) diluted in 10 mM HEPES, 0.05% Tween-20, 0.1 mM CaCl_2_, pH 7.2 to WGA-HRP (6.25–1.9 × 10^–5^ μM) was analyzed by loading the samples in standard capillaries (Cat# MO-K022, NanoTemper Technologies) and analyzing in the MST (Monolith NT.115, NanoTemper) using power (60%), excitation power (50%) at excitation 460–480 nm and emission 515–530 nm. Fluorescence signal changes were measured and data analyzed for kinetics using the MO. Affinity Analysis v2.3 software. For each sample, the fluorescence was averaged over 1 s before heating (*F*_cold_) and 19–20 s after heating (*F*_hot_). The normalized fluorescence (*F*_norm_) was calculated using the formula , and the *F*_norm_ for each concentration was fitted with a nonlinear regression model.^20^ The same procedure was followed to assess the binding between FITC-heparin and either AuNP-MSA or AuNP-WGAHRP. The concentration of gold nanoparticles was determined by estimating the molecular weight of nanoparticles. The volume of spherical nanoparticles was calculated from particle diameter (via TEM), and the number of gold atoms able to fit in this volume was calculated from the volume of a gold atom and adjusted for face centered cubic packing (74%). This was converted to molarity by the concentration of elemental gold in each sample as measured by ICP-MS. See Supporting Information for more details. The final sample concentration range was estimated to be 1.63 × 10^–1^–4.99 × 10^–6^ μM for AuNP-MSA and 1.98 × 10^–1^–6.05 × 10^–5^μM for AuNP-WGAHRP.

### Protein Preparation for Mass Spectrometry

A protein sample (5 μg) was reduced with dithiothreitol (DTT) at a final concentration of 10 mM for 10 min in boiling water. Following this, iodoacetamide (IAA) was added to a final concentration of 25 mM and incubated at room temperature for 20 min. Ammonium bicarbonate was then added to a final concentration of 40 mM, before 0.15 μg of sequencing grade modified trypsin (Promega, Cat. no. v5111)) was added to digest the proteins. Digestion proceeded for 16 h at 37 °C, after which samples were stored at −80 °C until analysis.

### Mass Spectrometry

Digested peptides were separated by nanoLC using an Ultimate nanoRSLC UPLC and autosampler system (Dionex, Amsterdam, Netherlands). Samples (2.5 μL) were concentrated and desalted onto a micro C18 precolumn (300 μm × 5 mm, Dionex) with H_2_O:CH_3_CN (98:2, 0.1% TFA) at 15 μL/min. After a 4 min wash, the precolumn was switched (Valco 10 port UPLC valve, Valco, Houston, TX) into line with a fritless nano column (length 75 mm, inner diameter 25 mm) containing C18AQ media (particle size 1.9 μm, pore size 120 Å Dr Maisch, Ammerbuch-Entringen, Germany). Peptides were eluted using a linear gradient of H_2_O:CH_3_CN (98:2, 0.1% formic acid) to H_2_O:CH_3_CN (64:36, 0.1% formic acid) at 200 nL/min over 30 min. High voltage (2000 V) was applied to low volume Titanium union (Valco) and the tip positioned ∼0.5 cm from the heated capillary (*T* = 275 °C) of a Orbitrap Fusion Lumos (Thermo Electron, Bremen, Germany) mass spectrometer. Positive ions were generated by electrospray, and the Fusion Lumos was operated in data-dependent acquisition mode (DDA).

A survey scan *m*/*z* 350–1750 was acquired in the orbitrap (resolution = 120,000 at *m*/*z* 200, with an accumulation target value of 400,000 ions) and lockmass enabled (*m*/*z* 445.12003). Data-dependent tandem MS analysis was performed using a top-speed approach (cycle time of 2 s). MS2 spectra were fragmented by HCD (NCE = 30) activation mode, and the ion-trap was selected as the mass analyzer. The intensity threshold for fragmentation was set to 25,000. A dynamic exclusion of 20 s was applied with a mass tolerance of 10 ppm. The resulting peptides were identified and matched to proteins via Mascot software v2.8.3 using the SwissProt database (2024_05) with a fragment ion mass tolerance of 0.4 Da and a parent ion tolerance of 5.0 ppm. Scaffold version 5.3.3 (Proteome Software Inc., Portland, OR) was used to validate MS/MS based peptide and protein identifications. Peptide identifications were accepted if they could be established at greater than 95.0% probability by the Peptide Prophet algorithm^21^ with Scaffold delta-mass correction. Protein identifications were accepted if they could be established at greater than 95.0% probability and contained at least 2 identified peptides. Protein probabilities were assigned by the Protein Prophet.^22^ Proteins that contained similar peptides and could not be differentiated based on MS/MS analysis alone were grouped to satisfy the principles of parsimony. Proteins were annotated with GO terms from NCBI (downloaded 23 Oct. 2024).^23^

## Results and Discussion

**Figure 1:**
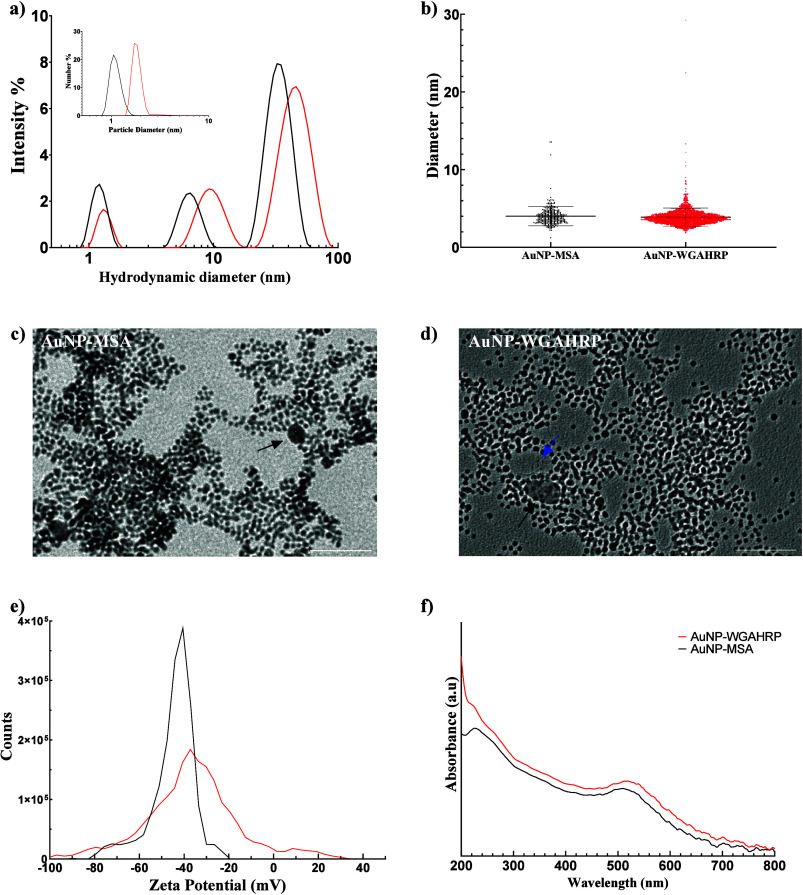
Physical characterization of AuNP–MSA (black traces) and AuNP–WGAHRP (red traces): a) MADLS hydrodynamic diameter by intensity distribution and number distribution (inset). b) Size measurements of AuNP–MSA (*n* = 323) and AuNP–WGAHRP (*n* = 2328) via TEM, lines and error bars represent mean and standard deviation. Representative TEM images of c) AuNP–MSA and d) AuNP–WGAHRP. Black arrows indicate larger size particles. Scale bar represents 50 nm. e) Zeta potential. Traces are representative of triplicate reads. f) UV–vis spectrum of AuNP–MSA and AuNP–WGAHRP.

AuNP–MSA and AuNP–WGAHRP were characterized by MADLS, NTA, TEM, zeta potential, and UV–vis spectroscopy. Three populations were detected by MADLS of hydrodynamic diameters 1.4, 6.6, and 30.9 nm for AuNP–MSA, which slightly increased to 1.5, 9.6, and 43.3 nm for AuNP–WGAHRP due to the conjugation of WGAHRP (Figure 1a). NTA demonstrated peak hydrodynamic diameters for AuNP-MSA and AuNP-WGAHRP to be 30.0 and 36.5 nm, respectively (Figure S1a, Table 1), which is in agreement with MADLS results. By TEM analysis, the diameter of AuNP cores for both samples were not significantly different (*p* > 0.05, two-tailed *t* test), averaging 4.01 and 3.87 nm, respectively (Figure 1b). Figure 1c and 1d display representative TEM images of AuNP-MSA and AuNP-WGAHRP. Some larger particles with diameters in the range of 20–30 nm are visible (black arrows), which may account for the 30.9 and 43.3 nm peaks detected in MADLS. The population of particles of ∼1.5 nm hydrodynamic diameter by MADLS are likely to be gold nanoclusters with core sizes significantly smaller than 1.5 nm. Some possible nanoclusters are indicated in Figure 1d (blue arrows); however, at this size, they are difficult to distinguish from background. The AuNP–WGAHRP samples were more uniformly distributed on the TEM grids than the AuNP–MSA (Figure 1c and d, respectively), indicating a higher colloidal stability. The TEM particle size of AuNP–WGAHRP aligns with previous studies.^1,4^ MADLS results show three peaks, which in the past was reported to be one peak. This was due to the difference in reporting. Previous studies reported z-average, which assumes a monomodal population (cumulants fit, weighted intensity average), while we have reported intensity and number-average distributions, which can account for multimodal distributions (distribution analysis, non-negatively least-squares fit). The discrepancy between TEM size and MADLS size is due to the conjugation of WGAHRP to AuNP-MSA. MADLS measures the hydrodynamic radius, which includes the hydrated MSA and protein layer coating the AuNP, while the hydration layer is lost in conventional TEM imaging due to drying of the sample. The zeta potential of the nanoparticles shifted slightly from −43.66 to −37.20 mV after conjugation of WGAHRP to the AuNP-MSA (Figure 1e). The peak broadening may reflect heterogeneous conjugation. The absorbance peak of AuNP–WGAHRP was also slightly red-shifted compared to AuNP–MSA (514 vs 504 nm, respectively). Uncapped AuNP (i.e., pure gold) contains no nitrogen or sulfur. MSA contains sulfur (21.3 wt %) but no nitrogen. WGAHRP contains both sulfur and nitrogen (3 and 15 wt % respectively. Using elemental analysis, we confirmed the conjugation of MSA by the presence of sulfur in AuNP-MSA (1.55 mg/L), and we confirmed the conjugation of WGAHRP to AuNP-MSA by the presence of nitrogen (4.47 mg/L) and the increased concentration of sulfur (2.46 mg/L) (Table 1). From TEM particle size and gold elemental analysis, the concentration of AuNP–MSA and AuNP–WGAHRP nanoparticles were calculated to be 0.33 and 0.4 μM, respectively. From sulfur analysis of AuNP-MSA, the number of MSA molecules per AuNP was calculated to be 148:1. From sulfur and nitrogen elemental analysis and amino acid composition analysis, the concentration of WGAHRP in AuNP–WGAHRP was determined to be between 0.43 and 0.45 μM (sulfur and nitrogen basis, respectively). Therefore, the number of WGAHRP particles per AuNP is 1.125:1 (nitrogen basis, see Supporting Information for calculations). A summary of the physical properties are listed in Table 1. We investigated the possibility of storing AuNP–WGAHRP in lyophilized form. Two freezing rates were trialled: snap freezing in liquid nitrogen or slow freezing in a −80 °C freezer at a controlled rate of −1 °C/min (see Supporting Information for details). After lyophilization, both samples were able to be reconstituted back into a stable suspension. By MADLS analysis, the slow-frozen sample had a similar hydrodynamic size as before freezing, while the snap frozen sample was significantly larger (Figure S1b, Table S1). Therefore, for long-term storage of AuNP-WGAHRP, we recommend lyophilization, with a freeze rate of −1 °C/min.

**Table 1:** Summary of Physical Properties of AuNP–MSA and AuNP–WGAHRP^a^

		AuNP–MSA		AuNP–WGAHRP	
		Intensity average	Number-average	Intensity average	Number-average
Particle Diameter (nm)	MADLS (nm)	30.9 ± 4.0	0.9 ± 0.4	43.3 ± 2.8	1.5 ± 0.3
		6.6 ± 0.6		9.1 ± 1.1	
		1.4 ± 0.2 (*n* = 3)		1.5 ± 0.3 (*n* = 3)	
	NTA (nm, mode ± standard error)	30 ± 0.3 (*n* = 5)		36.5 ± 0.4 (*n* = 5)	
	TEM (nm)	4.0 ± 1.2 (*n* = 323)		3.87 ± 1.2 (*n* = 2328)	
Zeta potential (mV)		–43.66 ± 1.20 (*n* = 3)		–37.20 ± 1.42 (*n* = 3)	
Absorbance peak (nm)		506		514	
Nitrogen conc. (mg/L)		0		4.47	
Sulfur conc. (mg/L)		1.55		2.46	
Gold conc. (mg/L)		83.90		83.74	
WGAHRP conc. (μM) (via elemental analysis)		-		0.45 (Nitrogen basis)	
		-		0.43 (Sulfur basis)	
AuNP conc. (μM) (via gold elemental analysis)		0.33		0.40	

a Values represent mean and standard deviation unless stated otherwise.

**Figure 2:**
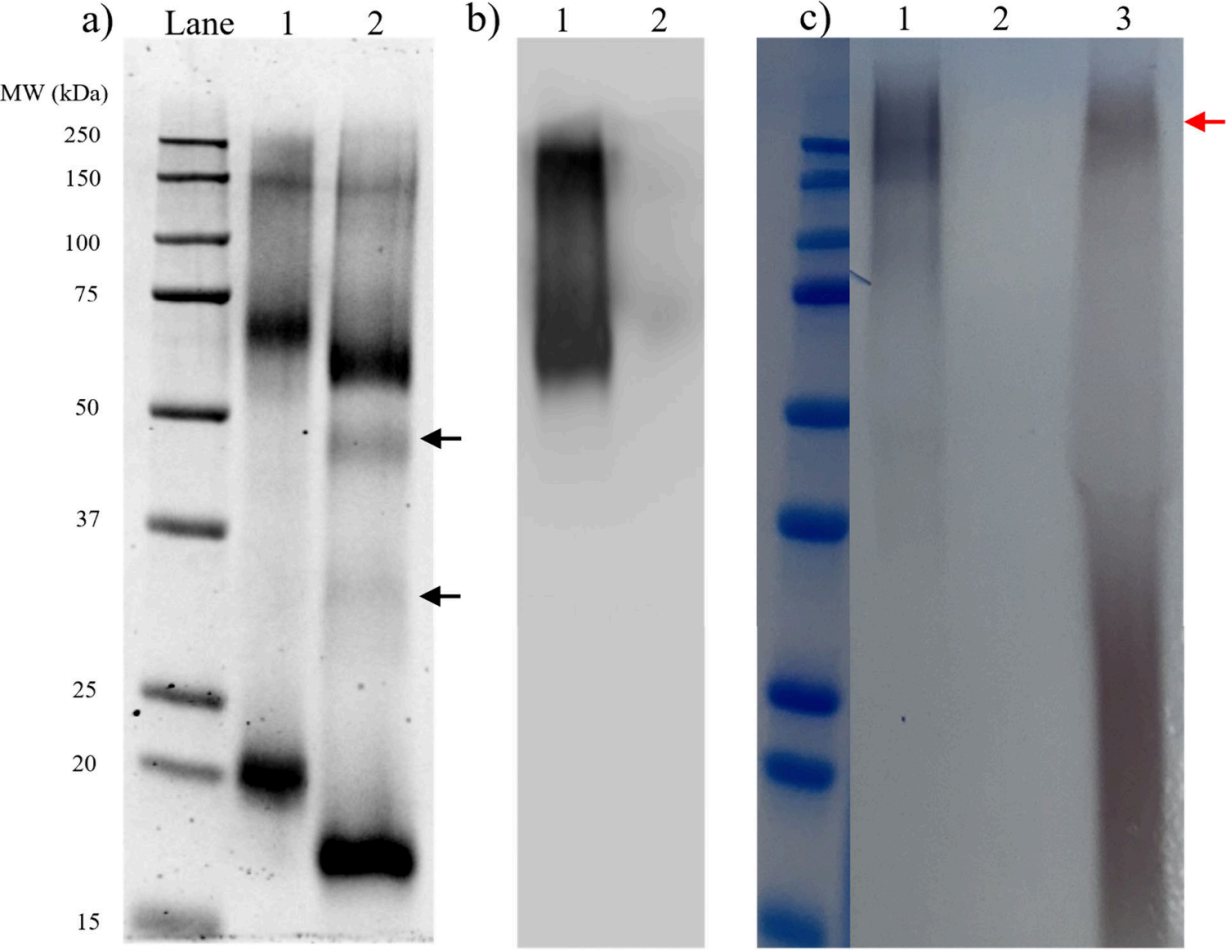
a) SDS-PAGE characterization of WGAHRP: neat (lane 1) and reduced (lane 2). New bands generated by reduction denoted by black arrows. b) Western blot characterization of WGA: neat (lane 1) and reduced (lane 2). c) SDS-PAGE characterization of AuNP–WGAHRP (lane 1), supernatant of precipitated AuNP–WGAHRP (lane 2), and AuNP–MSA (lane 3). High molecular weight gold band denoted by red arrow.

We characterized the WGAHRP stock material by SDS-PAGE (Figure 2a, Lane 1), which ran as three molecular weight bands, ∼150, 67, and 20 kDa, corresponding to two different sized WGAHRP conjugates and WGA monomer, respectively. This was surprising, as it was assumed that this conjugated material was in the form of 2WGA:1HRP, which should result in a size of ∼82 kDa. The ∼67 kDa band roughly corresponds to a 1WGA:1HRP ratio. The ratio of WGA:HRP in the ∼150 kDa band is difficult to determine, but it is likely a heterogeneous mixture of conjugates which lie in the range of 130–160 kDa. The denaturing conditions of the SDS-PAGE were able to separate WGA from the peroxidase conjugate as a 20 kDa band, which constituted 50% of the band intensity for the sample (remaining band intensities were ∼31% for the 67 kDa band and 18.3% for the 144 kDa band). WGA was expected to run as a 18 kDa band. A gain of 2 kDa indicates an association of WGA with several stabilizing GlcNAc (221.2 g/mol) and/or sialic acid (309.27 g/mol) saccharides. To probe this further, we reduced the WGAHRP with TCEP, which resulted in a molecular weight reduction in all bands. The WGA monomer (20 kDa) band dropped to ∼18 kDa, which indicates that reduction of WGA results in loss of tertiary structure and subsequent loss of ligands. According to the manufacturer’s Web site, a *meta*-maleimidobenzoyl-*N*-hydroxysuccinimide ester method was employed to construct this conjugate,^24,25^ which presumably links maleimide to thiol groups on WGA and ester groups on HRP. Two main possibilities exist for the existence of the 20 kDa band – a) WGA is predominantly bound to HRP glycan structures, of which there are 8,^17^ which can be disrupted by SDS, instead of covalently via thiol conjugation, or b) that for each WGA dimer, only one monomer is conjugated to HRP via thiol bonds, and thus WGA dimers are dissociated by SDS, resulting in a monomer band. Scenario a) is unlikely since maleimide and *N*-hydroxysuccinimide chemistry is robust. Scenario b) is possible since the WGA has been reported to run as a ∼18–20 kDa band previously.^26,27^ After reduction, the high molecular weight band marginally decreased; however, the gel resolution at this range is not sufficient to accurately determine the reduced molecular weight. The 67 kDa band was reduced to 60 kDa, while two fainter molecular weight bands appeared at ∼46 and 32 kDa (black arrows, Figure 2a, Lane 2). The 46 kDa band corresponds to fully glycosylated HRP containing the catalytic heme, while the identity of the 32 kDa band is unclear—at the gel resolution, this could correspond to the WGA dimer (36 kDa, this is unlikely due to SDS disruption as discussed earlier) or the HRP core protein (33.9 kDa). The retention of the high molecular weight bands after reduction may indicate incomplete reduction or perhaps preferential reduction of WGA before the WGAHRP thiol bond. Figure 2b shows a Western blot of replicate samples of Figure 2a, with Lane 1 containing WGAHRP and Lane 2 containing reduced WGAHRP. Since HRP was already present, no further probes were required and could be directly detected with chemiluminescent agents. This revealed two bands in Lane 1 of Figure 2b, one between 150 and 250 kDa and one at ∼65 kDa, which confirms that these two bands contain HRP. The 20 kDa WGA monomer band was not detected by chemiluminescence, since no other probes were used. Densitometry revealed that the amount of HRP between the 150 kDa band and the 67 kDa band were similar (∼47% to 53%, respectively). It then follows that the 150 kDa band is likely to consist of one HRP and 5–6 WGA monomers. Reduced WGAHRP in Lane 2 of Figure 2b was also not detected, indicating that the catalytic activity of HRP was destroyed by the reduction process.

To check the conjugation of WGAHRP to AuNP, we employed SDS-PAGE, resulting in a broad smear centered around 250 kDa (Figure 2c, Lane 1), which is significantly higher than the 150 kDa band of the neat WGA-HRP, and also two much fainter bands at ∼100 and 50 kDa. It is unclear what these bands correspond to. To check for unconjugated WGAHRP, we used the same amount of AuNP–WGAHRP as in Lane 1, precipitated it using a saturated solution of CaCl_2_ and MgCl_2_ and subjected the supernatant to electrophoresis (2.5 μg) (Figure 2c Lane 2). No bands were detected. The reported lower limit of detection and lowest practical sensitivity for this Coomassie stain is ∼4 and 15 ng, respectively,^28^ and so we conclude that the conjugation efficiency is at least >99.6%. As a control, we also ran a matched amount of AuNP–MSA in lane 3 of Figure 2. This also revealed a light band at approximately 200 kDa (red arrow, Figure 2c Lane 3) and a much lower streak from 15 to 37 kDa. Some gel deformation is visible just above the 37 kDa ladder marker. These gold nanoparticles were not able to be imaged effectively by the Coomassie filter (not shown), so were instead imaged in color (Figure 2c). By visual inspection, AuNP–MSA was not stained with Coomassie at all and presented as red streaks (Figure 2c Lane 3), while AuNP–WGAHRP was stained blue and presented as a blue-black color (Figure 2c Lane 1), which indicates the coincidence of AuNP with protein. Therefore, it appears that all molecular weight forms of the WGAHRP are conjugated to gold nanoparticles, as no separate protein-only stains were visible in the gel. The presence of a high molecular weight band in the AuNP–MSA indicates nanoparticles migrate to the same position as a ∼200 kDa protein. Interestingly, the theoretical molecular weight of ∼4 nm gold nanoparticles (modeled as a sphere with face centered cubic packing) is approximately 250 kDa (see Supporting Information). This may suggest that gold nanoparticles are able to migrate at their true molecular weight in SDS-PAGE gels. Accordingly, 15–37 kDa AuNPs correspond to a diameter of 1.5–2 nm, which were detected in MADLS but not TEM.

**Figure 3:**
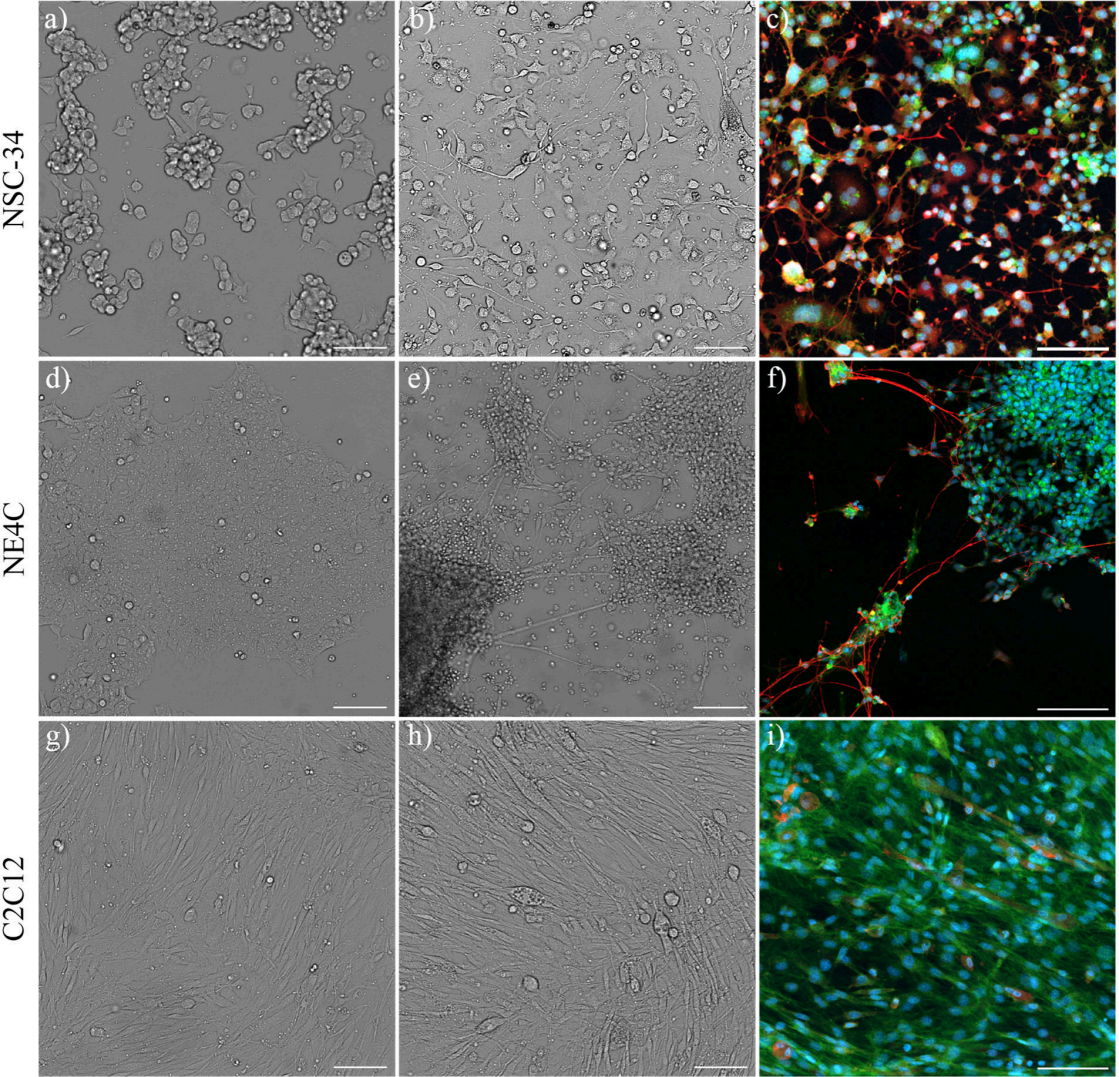
Microscopy images of NSC-34 (a-c), NE-4C (d-f), and C2C12 (g-i) cells grown under growth conditions (a, d, g), differentiation conditions (b, e, h) or stained with WGA-HRP488 (green), nuclei (blue) and differentiation markers (red) neurofilament 200 (c,f) or nicotinic acetylcholine receptors (i). Scale bar indicates 100 μm.

Next, we moved to characterize the cell lines we utilized as sources of glycoprotein. We chose these cell lines based on an intramuscular delivery, such that a WGA-mediated retrograde drug delivery vehicle would first encounter muscles, motor neurons and finally brain neurons. Neuroblastoma x spinal cord-34 (NSC-34) cells are a hybridoma cell line consisting of a fusion of neuroblastoma (N18TG2) and embryonic mouse (day 12–14) motor neuron-enriched spinal cord cells.^29^ In their growth state, they appear as a mixed population of clumped, circular colonies interspersed with more spread and irregularly shaped cells (Figure 3a), which possess the ability to spontaneously grow long processes. Differentiation of NSC34 cells occurs very rapidly on a laminin coated surface and serum free media (within 24 h). In their differentiated state, a much higher proportion of cells exhibit very long convoluted axons, frequently with *en-passant* boutons, and tend to form a convoluted network with other cells (Figure 3b). NSC34 cells were also able to differentiate on fibronectin and perlecan coated wells, but not collagen IV (Supplementary Figure S2).

Neuroepithelial-4C (NE4C) cells are derived from the cerebral vesicles of a p53 knockout 9 day-old mouse embryo.^30^ NE4C cells tend to grow in a much more highly clustered monolayer state (Figure 3d), with an exceptionally high growth rate compared to NSC34. Under differentiation conditions, the vast majority of NE4C cells die off, with the remaining adherent cells displaying two main phenotypes—the first being circular, highly clumped into spheroids, extending relatively straight axons; and a second phenotype, being extremely flat and spread (Figure 3e). Both phenotypes were capable of replication, as they were able to recover in number after the mass cell death, albeit much more slowly compared to their growth state. As the spheroid population grows, any existing axons that protrude outward begin to be suspended in the medium.

C2C12 are muscle cells isolated from the thigh muscle of dystrophic C3H mice and established by serial passage.^31^ The C2C12 cells also replicate extremely quickly but are bigger and spindle shaped (Figure 3g). In the differentiated state they tend to align directionally, elongate and fuse to form much larger cells, with some multilayer growth (Figure 3h). Differentiated NSC34 and NE4C cells stained strongly positive for neural marker neurofilament 200 (red, Figure 3c and f respectively), while differentiated C2C12 cells stained positive for myotube marker nicotinic acetylcholine receptor (red, Figure 3i). All cells stained strongly with WGA-HRP488. IgG antibody isotype staining revealed low intensity nonspecific staining in all three differentiated cell lines (Supplementary Figure S3).

Cell lines in the growth state are more representative of an embryonic/early life stage, while cell lines in their differentiated state are more representative of a mature stage. To purify the glycoproteins of these three cell lines in their growth and differentiated states, cell cultures were lysed with RIPA buffer supplemented with protease inhibitors. Interestingly, the cell lysate pellets from the NSC34 cells appeared visually different from the other cell lines, being almost transparent and resistant to pelleting via centrifugation, whereas the NE4C and C2C12 lysates pelleted as expected. For each of the three cell types, WGA-binding glycoproteins were purified from the cell lysates from one T25 flask of cells in growth condition (hereon designated NSC34-G, NE4C-G and C2C12-G) and three biological replicate T25 flasks of cells in differentiation conditions (hereon designated NSC34-D, NE4C–D and C2C12-D). This was because large numbers of morphologically similar cells could be obtained in the growth condition, while in the differentiation condition less cells could be obtained from one flask. Additionally, it was unclear if the differentiation process would introduce heterogeneity between replicates. Samples were assessed for variability by SDS-PAGE (Figure S4). The glycoprotein profiles between growth and differentiated states were similar with small variations. The profiles between the replicates for differentiated state glycoproteins were also similar. From one T25 flask, between 2 and 4 mg of protein was isolated from lysate, from which an estimated 5–20 μg of glycoproteins were extracted.

**Figure 4:**
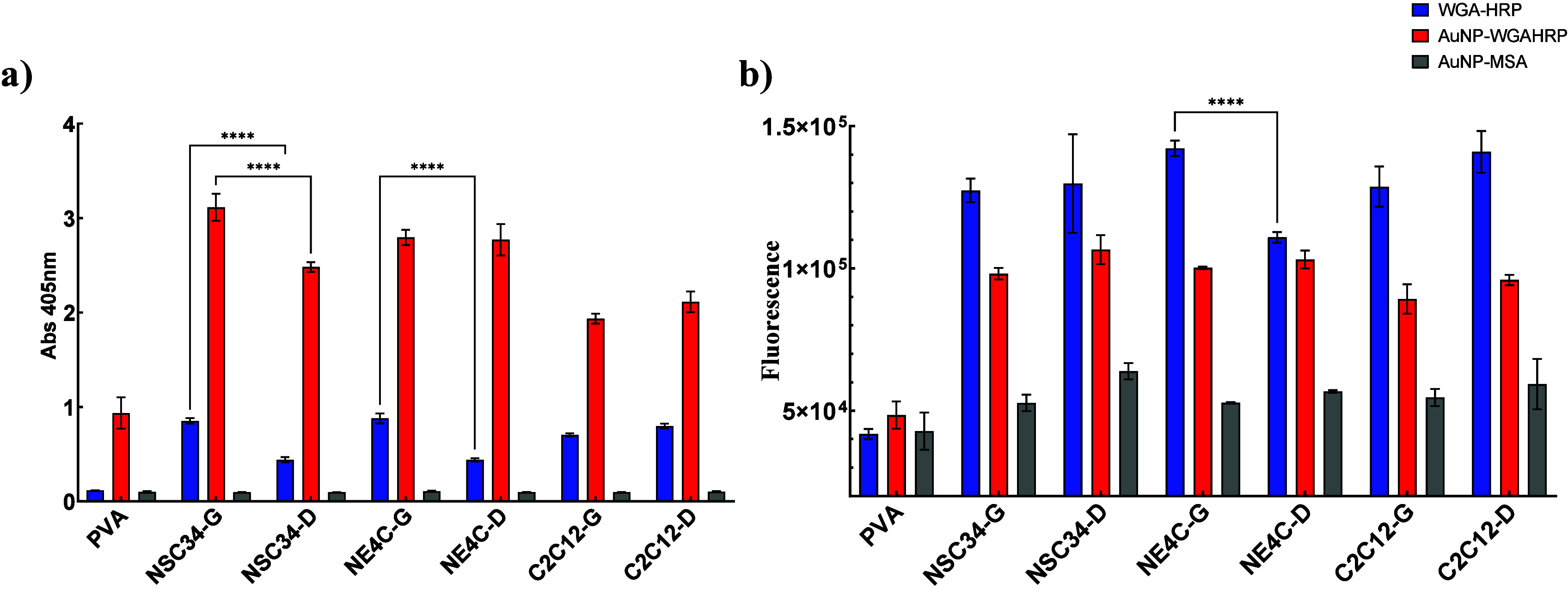
a) Enzyme linked lectin assay comparing the binding of WGAHRP (blue bars), AuNP–WGAHRP (red bars) and AuNP–MSA (gray bars) to purified glycoproteins from undifferentiated (G) and differentiated cells (D) from NSC-34, NE4C and C2C12 cell lines. b) Matched fluorescence-linked immunoassay using antibodies against WGA in the same plate. Data represents mean and standard deviation, *n* = 3. Significant differences between probes against matched cell lines denoted as asterisks (****, *p* < 0.0001) by two-way ANOVA.

We tested the ability of WGAHRP and AuNP–WGAHRP to bind to purified cell glycoproteins via an enzyme linked lectin assay (ELLA) (Figure 4a). Due to low yield, glycoproteins from biological triplicate differentiation conditions were pooled into one sample. Glycoprotein samples were immobilized on high-binding 96-well plates, blocked with poly(vinyl alcohol) (PVA) and probed with WGAHRP or AuNP–WGAHRP (matched WGAHRP concentrations of 1 μg/mL). PVA was used as a negative control and blocking agent instead of BSA, as we found that WGAHRP was able to bind to BSA-blocked surfaces (Figure S4), as reported elsewhere,^32^ making it unsuitable as a blocking agent in this experiment. There was a high nonspecific signal for AuNP–WGAHRP binding to PVA coated surfaces. Even after correcting for this nonspecific signal, the signal for AuNP–WGAHRP (red bars) was 2–5 times higher than that of WGAHRP (blue bars) for all glycoprotein samples. For NSC-34 glycoproteins, the absorbance reading was higher for glycoproteins from the growth state (NSC-34G) than differentiation state (NSC-34D) for both WGAHRP and AuNP–WGAHRP detection. For NE4C cells, the absorbance reading was higher for WGAHRP based detection of glycoproteins from the growth state (NE4C-G) compared to the differentiated state (NE4C–D), but not for AuNP–WGAHRP detection. For C2C12 cells, the absorbance readings were not significantly different between growth state and differentiated states for either WGAHRP or AuNP–WGAHRP detection. As a control, we probed all samples with AuNP–MSA, which did not generate any signal. From this we conclude that more WGAHRP binds to glycoproteins from nerve cells in the growth state than in the differentiated state. In contrast, WGAHRP binds similarly to glycoproteins from muscle cells in either growth or differentiated state. Even after accounting for nonspecific binding, AuNP–WGAHRP generated a stronger signal than WGAHRP (approximately 2–3 times stronger). The colorimetric agent used was ABTS, which is converted from a colorless form to a green color in the presence of a peroxidase reaction. From this we hypothesize that there is an accelerated H_2_O_2_ decomposition mediated by AuNP, while AuNP itself has no inherent catalytic activity toward H_2_O_2_. In order to test the hypothesis of AuNP-mediated acceleration of H_2_O_2_ decomposition, we probed the exact same samples in the same plate with WGA antibodies in a fluorescence-linked immunoassay (FLISA) (Figure 4b). This revealed an opposite trend to the ELLA (Figure 4a), showing that more WGAHRP was bound to immobilized glycoproteins than AuNP–WGAHRP for all samples (blue bars vs red bars respectively), except NE4C–D for which the signal intensities were not significantly different. This supports the hypothesis of AuNP-mediated acceleration of H_2_O_2_ decomposition. Out of the three cell lines, only NE4C showed a significant difference between WGAHRP binding to growth and differentiated glycoproteins, in which WGAHRP bound more to growth glycoproteins than differentiated glycoproteins (Figure 4b, blue bars, NE4C-G > NE4C–D). This was not the case for AuNP-WGAHRP, which did not show any significant differences between growth and differentiated glycoproteins for any cell lines. After correction for nonspecific binding to PVA, the fluorescent intensity for AuNP–WGAHRP binding to glycoproteins was approximately 50% lower than that for WGAHRP over all the samples, except for NE4C–D which had a similar fluorescence intensity between the two probes. This means that after differentiation, NE4C cells may stop expressing a high-affinity WGA-binding structure. From differences in fluorescence intensity, we posit that the enzyme-linked lectin assay is more accurate for detection of WGAHRP binding, but it is hard to compare directly with the AuNP–WGAHRP conjugate due to the signal amplification. Comparatively, the FLISA allows a better comparison between the conjugated and unconjugated WGAHRP; however, it did not replicate most of the differences seen in the ELLA, and so is less sensitive. In an attempt to probe the signal amplification by AuNP–WGAHRP binding, we increased the concentration of WGAHRP by half, and reduced the concentration of AuNP–WGAHRP 7 times (Figure S4). At these concentrations, the signal from WGAHRP binding to NSC34-G, NE4C-G, C2C12-G and -D glycoproteins was double that of AuNP-WGAHRP. However, for NSC34-D and NE4C–D samples, the signal intensity was roughly similar between AuNP–WGAHRP and WGAHRP. Since the binding patterns are different between conjugated and unconjugated WGAHRP, and between certain glycoprotein samples, we conclude that conjugation of WGAHRP to AuNP does change binding affinity, although we may not quantify it through this method. In a previous paper, we have shown that the retrograde transport behavior and velocity of AuNP-WGAHRP-containing endosomes is similar to WGAHRP-containing endosomes.^4^ Together this indicates that while the WGAHRP conjugation does reduce binding affinity slightly, it does not affect the retrograde transport characteristics. This is likely due to the multiple ligand binding sites of WGA (8 sites for GlcNAc and 2 for sialic acid per WGA dimer),^5,6^ which allows for redundancy in ligand binding.

The use of glycoproteins from cells in the growth state and the differentiated state are reflective of glycoprotein expression changes from embryonic to mature states, respectively. In the context of WGAHRP ligand binding, the significance of this is difficult to know, as the glycan structures associated with these different glycoproteins samples was not determined. However, from WGAHRP binding data (Figure 4a, blue bars) we can broadly conclude that nerve cells in the growth state (embryonic-like) contain more WGA-binding glycan structures and/or glycan structures with higher affinity than the differentiated state (mature-like), while for muscle cells this does not change significantly. In comparing the binding differences between unconjugated and conjugated WGAHRP (blue bars vs red bars respectively) to growth and differentiated glycoproteins, in the NE4C samples we observed significant differences (WGA: growth > differentiation, AuNP-WGAHRP: growth = differentiation), but not in the NSC34 (WGAHRP and AuNP-WGAHRP: growth > differentiation) or C2C12 samples (WGAHRP and AuNP-WGAHRP: growth = differentiation). It follows that the conjugation event affects binding only in some cases (i.e., in NE4C growth state glycoproteins). There are two likely reasons for these differences: obscuration of binding sites or disruption of binding sites by conjugation. The former is a more straightforward phenomenon, where AuNP physically blocks binding sites. The latter is a more complicated scenario involving sterics of binding. It has been shown that spacing between ligands is critical for lectin binding affinity. For example, in a glycan array, no binding was observed for monomeric GlcNAc, whereas for dendrimerized GlcNAc and densely packed GlcNAc gluconeopeptides binding was clearly seen.^33^ Elsewhere it is shown that the K_d_ value for WGA to GlcNAc trisaccharide is 4.7 μM, but for 3 repeating n-acetyllactosamine units (LacNAc, galactose-GlcNAc disaccharide), the K_d_ value is 550 μM.^34^ The extra spacing by galactose units between GlcNAc units causes a ∼100-fold decrease in binding affinity. Therefore, we envision a scenario where AuNP-WGAHRP conjugation causes a subtle change in the WGA presentation, such that out of a highly diverse pool of GlcNAc-containing glycan structures, a certain subset cannot be recognized, which in this case is expressed by the NE4C cell line.

The comparison of conjugated vs unconjugated WGAHRP binding to natural ligands was confounded by the presence of the AuNPs. With the ELLA, an acceleration of peroxidase reaction was observed, as compared to WGAHRP by itself. The peroxidase capability of AuNPs has been widely reported for colorimetric, fluorescent and chemiluminescent detection.^35,36^ ABTS, the colorimetric substrate used for detection in the ELLA, has been shown to be able to be autocatalytically oxidized by H_2_O_2_ at high concentrations (above ∼100 mM) in the presence of AuNPs, but the catalytic activity was much higher for bare AuNPs as compared to citrate-capped AuNPs (negatively charged).^37^ Other studies report the higher autocatalytic activity in acidic pH conditions. In this study, 0.03 v/v% (0.88 mM) H_2_O_2_ was used, which is outside of the range of the detection for the amount of time allowed for color development. Additionally, a phosphate-citrate buffer was used for ABTS which is known to suppress autocatalysis of H_2_O_2_.^38^ Therefore, the acceleration of the ABTS oxidation reaction is most likely to the direct-electron transfer effect, which has been well documented to occur between iron-containing hemes (the catalytic center of HRP) and AuNP.^39,40^ This implies the conjugation event brings the catalytic center close to the surface of AuNP. The matched comparison of the ELLA with FLISA provided another way to estimate any binding differences between WGAHRP and AuNP-WGAHRP, revealing a higher binding for WGA-HRP, which agrees with the initial hypothesis. However, the sensitivity of this assay was quite low, with a high gain setting required to achieve a good signal, and still did not fully replicate all the features of the ELLA despite the high concentration of primary and secondary antibodies used.

To further investigate whether the conjugation of WGAHRP to AuNP alters its binding properties, we used MST.^20^ We investigated the binding of WGA-HRP and AuNP-WGAHRP to a simpler ligand, heparin, the most highly sulfated natural glycan composed of repeating disaccharides of uronic acid and GlcNAc. Several other techniques exist that can probe the molecular interactions of two binding partners, such as surface plasmon resonance, bilayer interferometry, microscale thermophoresis and quartz crystal microbalance. Surface plasmon resonance was deemed to be unsuitable due to the inherent plasmon resonance of AuNPs. Bilayer interferometry was attempted, but binding signals were convoluted with plasmon resonance responses of AuNPs, resulting in a very poor signal-to-noise ratio (data not shown). MST was chosen for this application due to the small volume of substrate required.

**Figure 5:**
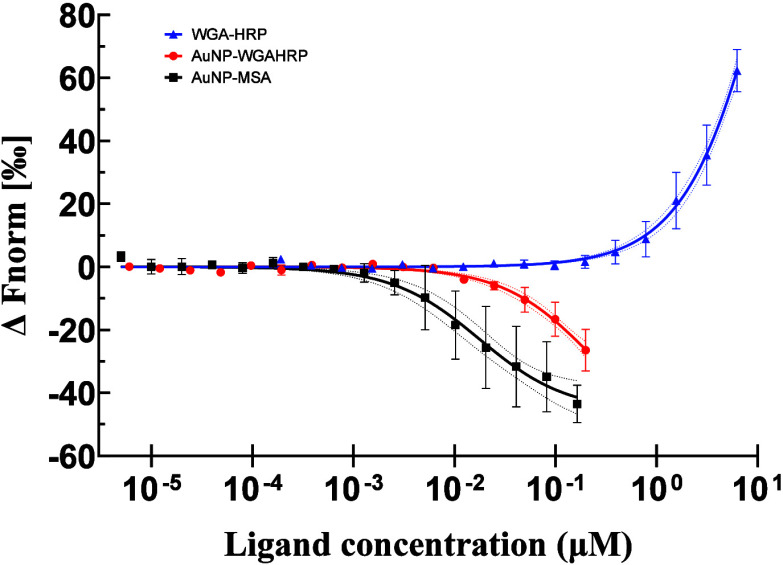
Fitted dose response curves of FITC-Heparin with WGA-HRP (blue), AuNP–WGAHRP (red) and AuNP–MSA (black). Data points and error bars represent mean and standard deviation respectively, *n* = 3. Associated 95% confidence intervals of the fitted curves are plotted as dotted lines.

The raw baseline MST response of FITC-heparin by itself is shown in Figure S7a, depicting the four phases of MST: I) Steady state fluorescence, II) Temperature jump (T-jump) directly after the heating IR laser is turned on; III) Diffusion-limited thermophoresis; IV) inverse T-jump, directly after the heating IR laser is turned off. We observed different changes in fluorescence for FITC-heparin when adding WGA-HRP, AuNP-WGAHRP, or AuNP-MSA, with the raw MST response at their highest concentrations depicted in Figure S7b. WGA-HRP binding to FITC-heparin yields higher normalized fluorescence changes (Δ*F*_norm_) with increasing WGA-HRP concentration with an estimated *K*_d_ of 17.64 ± 7.35 μM (Figure 5). The opposite trend was seen for AuNP-WGAHRP and AuNP-MSA resulting in a downward trending *F*_norm_ curve with increasing AuNP-WGAHRP/AuMSA concentration which resulted in estimated *K*_d_ of 0.22 ± 0.07 μM and 0.018 ± 0.01 μM, respectively. The raw MST traces for each condition are shown in Figure S5c-e.

We encountered an issue with strong fluorescence reduction with AuNP samples, which is the opposite of what we observed with WGAHRP (Figure 5). Interestingly, AuNP-MSA caused an even larger fluorescence reduction compared to AuNP-WGAHRP. It is difficult to determine whether this is due to nonspecific binding or merely energy transfer due to physical collision between molecules. The fact that it caused a larger fluorescence reduction than AuNP-WGAHRP may suggest that WGAHRP conjugation to the nanoparticle may increase the distance between the AuNP and FITC-heparin, thereby reducing quenching effects. Alternatively, this may indicate a unique conformation that presents an intermediate effect between the other two. It should be noted that while the dose response curves of the AuNP samples trend in the opposite direction from WGAHRP, the calculated *K*_d_ values are still valid, as the directionality is only indicative of decreased or increased fluorescence as compared to that of FITC-heparin alone.

Two main possibilities existed for this enhanced fluorescence reduction: a) the IR laser induces photothermal heating of the AuNPs bound to FITC-heparin, which enhances the rate of thermophoresis away from the heated volume, or b) AuNPs are able to quench fluorescence. Most reports of photothermal heating of AuNPs involve irradiation in the first NIR window between 700 and 950 nm,^41^ whereas the MST machine uses an IR laser at 1450 nm. Therefore, we exclude photothermal excitation of AuNPs as a cause of enhanced fluorescence reduction. There are many reports of metallic, and in particular AuNPs both quenching and enhancing fluorescence,^42^ showing a dependence on size, coating and the wavelengths of the excitation and emission light. The (likely) mechanism of quenching is known as nanometal surface energy transfer (NSET) and is analogous to Förster resonance energy transfer (FRET). NSET efficiency is inversely proportional to the fourth power of the distance between the metal and the fluorophore and is also sensitive to the spectral overlap between the absorbance of the metal and the emission band of the donor (fluorophore). Particle size is also known to play a role, with bigger particles providing stronger quenching due to larger plasmon field effects.^43^ A more in-depth theoretical discussion of NSET is available elsewhere.^44^ From the MST experiments (Figure 5), it is not possible to determine whether the quenching of the FITC-heparin by AuNP–MSA is due to nonspecific binding or due to collision events. Combined information from other techniques, fluorescence lifetime imaging (FLIM) and/or fluorescence correlation spectroscopy (FCS) could provide the information necessary to make this assessment. However, from the *K*_d_ values of AuNP–MSA and AuNP-WGAHRP, (0.017 and 0.22 μM, respectively) we may infer that a ∼10-fold increase in *K*_d_ value is indicative of an increase in distance by half the original distance (due to the inverse fourth power rule). Using a far red or infrared fluorophore in the future may avoid issues with quenching by avoiding the absorbance band of AuNPs. What is clear is that the AuNP–WGAHRP produces a distinctly different dose–response curve and has a lower NSET efficiency, which provides confidence to specific binding between AuNP–WGAHRP and FITC–heparin. Our measurement of the dissociation constant between WGAHRP and heparin (*K*_d_ = 17.64 μM) is the first of its kind to our knowledge. Interestingly, in a glycoconjugate microarray, WGA showed no binding to immobilized heparin GAGs,^34^ which may reflect flexible conformation requirements for binding. Measurements of binding affinity between WGA and other similar ligands by a variety of methods include: GlcNAc, *K*_d_ = 2.2 mM,^45^ 2.4 mM;^46^ GlcNAc tetrasaccharide, *K*_d_ = 81 μM,^46^ 90 μM,^46^ 4.1 μM;^34^ de-N-acetylated chitosan, *K*_d_ = 150 μM;^45^ LacNAc, *K*_d_ = 57 μM.^34^ Large variations in reported *K*_d_ values for the same ligand in the literature are reflective of the different methods of ligand immobilization and the critical but complicated ligand spacing requirements for lectin binding.

In the interest of identifying WGA-binding proteins capable of retrograde transport, glycoproteins samples were submitted to the mass spectrometry facility for protein identification. After filtering the list at a 95% probability threshold with a minimum of 2 detected peptides (see methods), a total of 1002 WGA-binding glycoproteins were identified with approximately 600–700 identified proteins per sample. The full list of identified proteins with the exponentially modified protein abundance index (emPAI, a method for quantitative estimation of protein abundance in mass spectrometry^47^) for each glycoprotein sample is available in the Supporting Information, annotated with Gene Ontology (GO) terms.^23,48,49^ As they were extracted from the cell lysate, these glycoproteins come from all regions of the cell and span a wide range of functions, from cell adhesion, protein folding, RNA transcription, endocytosis, transport of cargo between organelles to nuclear import. Between the growth and differentiated conditions for each cell type, there were between 71 and 182 differentially identified proteins, representing 10–25% of glycoproteins (Table 2). From these lists, there was no obvious trend as to why more WGAHRP binds to nerve cell growth-state glycoproteins more than differentiated-state glycoproteins, as was seen in Figure 4a.

**Table 2:**
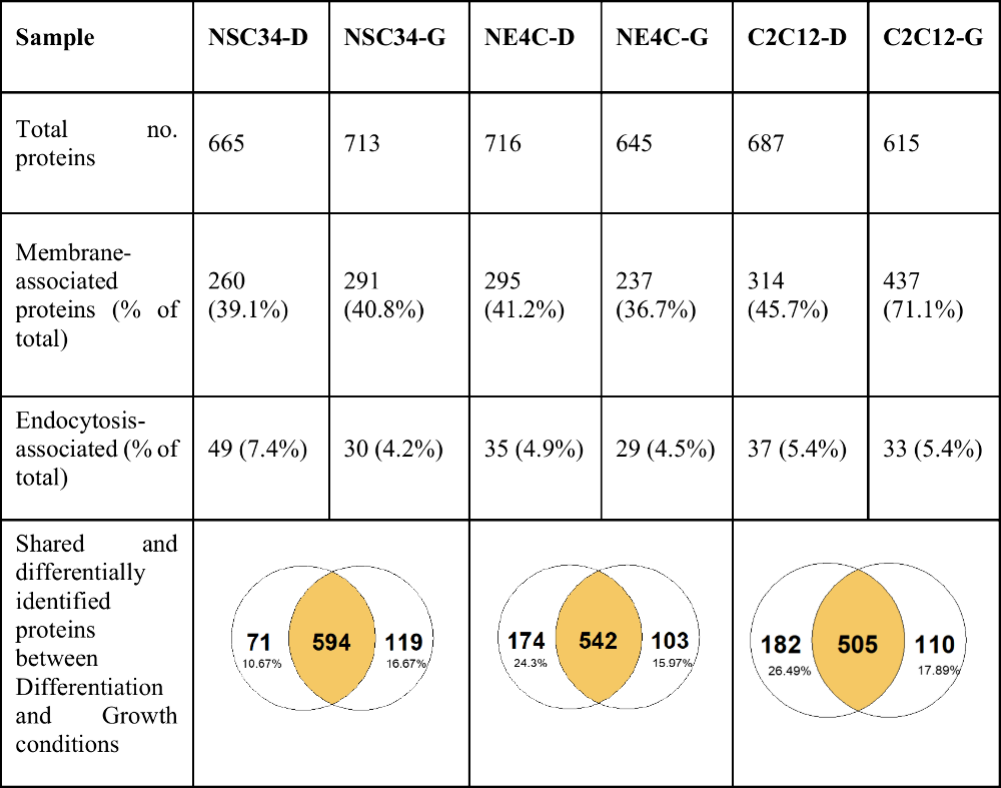
Summary of Identified Glycoproteins by Mass Spectrometry from NSC34, NE4C and C2C12 Cells in Differentiation Conditions (D) or Growth Conditions (G)

In the context of WGA-mediated retrograde transport, the membrane and endocytosis-associated proteins are the most relevant. Approximately 40% of identified proteins were annotated with the “membrane” description, while approximately 4–7% of proteins were annotated with the “endocytosis” description (Table 2).

From these identified proteins, their associated descriptive annotations and literature, we suggest 25 WGA-binding proteins that may be involved in retrograde transport of WGA (Table 3). Here we consider the first-order interaction of WGA with membrane-bound glycosylated proteins, which are subsequently endocytosed and sorted for retrograde transport. Second-order interactions, which would include events where WGA is indirectly endocytosed prior to retrograde transport (e.g., bound to another protein which binds to a receptor, causing uptake), are not considered, although we do not rule out this possibility. Indeed, quite many proteins were identified with high emPAI scores, which are not known to be glycosylated, such as calnexin, calreticulin, calmodulin, various tropomyosin chains and high mobility group protein 1 and 2, indicating that they are bound to and copurify with other glycosylated proteins as a complex. Membrane proteins involved with lysosome trafficking and catabolic processes were also not included, since proteins targeted in this manner are more likely to be degraded rather than transported between cells. Given their annotated functions, subcellular location and abundance in cell lines (by emPAI and % peptide coverage), we provide a qualitative ranking from high to low likelihood of being a retrograde transport binding partner for WGA (Table 3).

**Table 3:** Non-Exhaustive List of Possible Candidates for WGA-Binding Retrograde Transport Proteins^a^

		Sample emPAI score /% peptide coverage								
Candidate	Accession no.	NSC34-G	NSC34-D	NE4C-G	NE4C-D	C2C12-G	C2C12-D	Subcellular Location	Function	Candidate likelihood
Src substrate cortactin	Q60598	1.07/(24%)	1.30/(29%)	0.869/(25%)	1.07/(31%)	0.969/(23%)	0.44/(13%)	M	Cytoskeletal organization, receptor-mediated endocytosis, endosome recycling	High
Transmembrane protein 106B	Q80X71	0.223/(11%)	0.106/(4.7%)	0.106/(4.7%)	0	0.106/(4.7%)	0.223/(8.4%)	M, LM, LEM	Involved in axonal sorting and transport of late endosomes and lysosomes, dendritic	High
Amyloid-β precursor protein	P12023	0	0.117/(4.9%)	0	0.117/(4.5%)	0.038/(1.7%)	0.159/(6.6%)	M, EM	Neurite growth, neuroprotection, neurotransmitter release modulation	High
Golgi apparatus protein 1	Q61543	0.579/(18%)	0.697/(19%)	0.914/(22%)	1.38/(33%)	0.334/(11%)	0.505/(18%)	M	FGF and E-selectin binding, trafficking to Golgi apparatus	High
Trans-Golgi network integral membrane protein 1	Q62313	0.650/(29%)	0.182/(7.6%)	0.285/(20%)	0.182/(10%)	0.396/(14%)	0.285/(12%)	M, GM	Regulates membrane traffic between membrane and Golgi apparatus	High
Aminopeptidase N	P97499	0.0297/(2.7%)	0	0.0297/(2.2%)	0.0297/(1.7%)	0.228/(12%)	1.41/(36%)	M	Peptide processing and digestion	High
Synaptotagmin-1	P46096	0	0.143/(5.2%)	0	0.143/(8.6%)	0	0	M, SVM	Neurotransmitter release, synaptic vesicle trafficking at active zone	Medium-High
Prolow-density lipoprotein receptor-related protein 1	Q91ZX7	0	0	0.006/(0.31%)	0.019/(0.75%)	0.351/(12%)	0.576/(19%)	M	Cellular lipid homeostasis, extracellular protease degradation, promiscuous endocytic receptor	Medium-High
Low-density lipoprotein receptor-related protein	A2ARV4	0	0	0.064/(3.2%)	0.019/(1.1%)	0	0	M	Cellular lipid homeostasis, highly promiscuous endocytic receptor	Medium-High
Low-density lipoprotein receptor	P35951	0	0	0.069/(4.6%)	0.107/(6.3%)	0.107/(5.9%)	0	M, EM, LEM	LDL uptake	Medium-High
Heat shock protein HSP 90α	P07901	0.303/(12%)	0.255/(9.3%)	0.353/(12%)	0.303/(9.5%)	0.303/(12%)	0.353/(12%)	M	Molecular chaperone, protein folding, dynein assembly^50^	Medium-High
Heat shock protein HSP 90β	P11499	0.360/(15%)	0.413/(16%)	0.586/(18%)	0.259/(9.7%)	0.413/(18%)	0.586/(21%)	M	Molecular chaperone, protein folding, dynein assembly^50^	Medium-High
Neuronal pentraxin receptor	Q99J85	0.0627/(2.2%)	0.129/(9.5%)	0	0	0.0627/(2.2%)	0	M	Synaptic vesicle uptake, glutamate receptor clustering	Medium
Integrin β3	O54890	0.159/(8.1%)	0.038/ (1.9%)	0	0	0.117/5.50%	0.117/4.40%	M	Cell adhesion, FGF and IGF binding	Medium
Integrin β5	O70309	0.076/(3.1%)	0.076/(4.0%)	0.245/(9.1%)	0	0.157/(8.3%)	0.792/(23%)	M	Cell adhesion	Medium
Integrin αV	P43406	0.215/(9.6%)	0.087/(3.2%)	0.118/(5.9%)	0.285/(11%)	0.285/(12%)	0.65/(24%)	M	Cell adhesion	Medium
Basigin/CD147	P18572	0.959/(22%)	1.110/(25%)	1.850/(26%)	1.110/(25%)	0.566/(17%)	0.818/(24%)	M	Adhesion, matrix metalloprotease induction, nutrient transport^51^	Medium
Neural adhesion molecule 1	P13595	0.497/(17%)	0.207/(12%)	0.027/(2%)	1.5/(30%)	0.58/(17%)	1.12/(24%)	M	Cell-cell adhesion	Medium
Galectin 3-binding protein	Q07797	0.640/(30%)	0.811/(30%)	0	0.104/(6.8%)	0.281/(14%)	0.561/(24%)	E	Integrin mediated cell adhesion, Galectin 3 binding	Medium
Vesicular integral-membrane protein VIP36	Q9DBH5	0.265/(16%)	0.082/(2.5%)	0	0.082/(2.5%)	0.265/(8.7%)	0.265/(12%)	GM	Transport and sorting of glycoproteins in early secretory pathway	Medium
CD151 antigen	O35566	0.249/(15%)	0.249/(9.1%)	0	0	0.396/(9.1%)	0.744/(5.9%)	M	Component of specialized membrane microdomain for receptor clustering and signaling	Low-medium
CD44 antigen	P15379	0	0	0	0	0.206/(6.4%)	0.07/(3.6%)	M	Extracellular sensing, signal transduction	Low
Sortilin	Q6PHU5	0.036/(1.3%)	0	0	0.073/(2.5%)	0	0	M, ERM, LM, EM, NM, GM	Membrane to Golgi apparatus trafficking	Low

a M = membrane, LM = lysosome membrane, LEM = late endosome membrane, ERM = endoplasmic reticulum membrane, NM = nuclear membrane, EM = endosome membrane, GM = Golgi apparatus membrane, SVM = Synaptic vesicle membrane, E = excreted.

The mass spectrometric identification of WGA-binding retrograde transport partners is a novel attempt—the promiscuity of WGA binding among glycoproteins has likely prevented previous identification of the retrograde transport partners. These will require rigorous experimental testing for verification. While we have manually curated a nonexhaustive list of these binding potential partners, we acknowledge some limitations in this work. In future studies, to gain greater selectivity and ease analysis, membrane proteins should first be extracted (instead of whole cell lysate) from which glycoproteins should be subsequently purified. Deglycosylation before processing for mass spectrometric analysis would reveal more tryptic digestion sites, improving the number of peptides available for identification, in turn increasing the confidence of protein identification. Additionally, in the future, replicate samples should be analyzed to provide a better estimate of protein abundance, something which could not be done in this study due to scarcity of sample.

The molecular mechanisms of retrograde transport have been well studied. In general, cellular cargo (vesicles, endosomes, etc.) to be trafficked are attached to microtubule motor proteins: kinesins for anterograde transport (away from the nucleus) and dynein for retrograde transport (toward the nucleus). Dynein is recruited to the “plus” end (distal end with respect to the nucleus) of microtubules via recruitment of EB and CLIP-170 proteins to a glycine-rich domain known as CAP-Gly. The activity of dynein is regulated by binding of dynactin and lissencephaly-1 (LIS1), causing a conformational change to an “open” (active) configuration.^52,53^ This dynactin-dynein complex is anchored to microtubules by cargo-specific adaptors such as bicaudal D homologue 2 (BICD2)^54^ or Hook1,^55^ which promote motility from the “plus” end toward the “minus” end of microtubules (which are polarized in orientation). Some adapters allow for the binding of two dyneins per dynactin, increasing the force and speed of the complex.^56,57^ Currently there are several known dynein-activating adaptors, with several more candidates.^58^ An excellent overview of dynein and associated adaptors is given by Olenick et al.^59^

Combining the knowledge of retrograde adaptor proteins with the mass spectrometric data here may provide a good starting point for future studies in this area. Coimmunopurification combined with colocalization studies may reveal more unique binding partners. For a more direct proof of the retrograde-signaling binding partners for WGA, a next-generation spatial multiomic approach may provide a more powerful analysis, which allow multiplex readouts, combining proteomic data with genomic or RNA transcriptomic data mapped to their physical location on a sample. This may allow capturing of a nerve cell in the process of retrograde transport of WGA-containing endosomes. This would produce a highly accurate and relevant set of targets and allow the profiling of many more protein targets than traditionally possible. Genetic knockout of these targets in a retrograde transport assay will provide conclusive proof of their role.

## Conclusion

Our interest in the retrograde axonal transport phenomenon caused by the promiscuous binding of WGA to cell membrane glycoproteins led us to investigate the effect of AuNP-WGAHRP conjugation on binding to several native ligands. We have shown that our AuNP–WGAHRP conjugate is able to bind glycoproteins purified from three different cell lines in growth/embryonic and differentiated/mature states, and heparin. Binding was reduced for AuNP-WGAHRP conjugates compared to WGAHRP; however, quantification of this difference in binding by conventional biochemical methods (fluorescence, colorimetric) was hampered by some unique properties of AuNPs, namely direct electron charge transfer, which accelerates peroxidase reactions, and nanometal surface energy transfer, which, in the conditions tested, produced strong quenching of fluorophores. These same properties may be useful in other applications. A better understanding of structure–function relationships in protein-nanoparticle conjugates (i.e., how conjugation affects lectin binding affinity) will inform future work in this area. We have conducted proteomic analysis of the purified glycoproteins and tentatively suggest several candidate binding partners, which may induce retrograde axonal transport of WGA. These candidates include amyloid β precursor protein, transmembrane protein 106B, Src substrate cortactin, synaptotagmin-1, heat shock protein 90 and low-density lipoprotein receptor-related protein. Future work involving genetic engineering and multiomic spatial imaging can provide strong evidence for these. If verified, this will allow better targeting of therapies for nervous system disorders via neural tracing routes.

## Supporting Information Available

Additional figures showing NTA hydrodynamic particle size, particle size after reconstitution of lyophilized nanoparticles, phase contrast micrographs of nerve cell differentiation on different protein substrates, SDS-PAGE of purified glycoproteins, additional ELLA results, MST raw traces; list of chemicals, materials and suppliers (.docx) Full list of proteins identified by mass spectrometry for all glycoprotein samples (.xlsx) Conversion of elemental analysis to AuNP, MSA and protein concentrations (.xlsx) (ZIP)

## Notes

Representation of WGA in TOC figure generated with Mol* Viewer.^60^

## Abbreviations

WGA: wheat germ agglutinin
HRP: horse radish peroxidase
AuNP: gold nanoparticle
SDS-PAGE: sodium dodecyl sulfate-polyacrylamide gel electrophoresis
TEM: transmission electron microscopy
MADLS: multiangle dynamic light scattering
ELLA: enzyme-linked lectin assay
IF: infrared
FLISA: fluorescence-linked immunoglobulin assay
NSET: nanometal surface energy transfer
