# Supplementary material for: Interfacial Interactions between Neural Tracing Lectin–Gold Nanoparticle Conjugate and Cell Membrane Glycoproteins
Source: Langmuir. 2025 Apr 16;41(16):10161–76. doi: 10.1021/acs.langmuir.4c05034 (PMC12044695; doi:10.1021/acs.langmuir.4c05034)
Supplement: Supplementary file 1 — la4c05034_si_001.zip [file la4c05034_si_001.zip › Supplementary Information.docx]

Supplementary Information - Interfacial interactions between neural tracing lectin-gold nanoparticle conjugate and cell membrane glycoproteins

Dr. Joel Yong^1,*^, Dan Wang^2^, Lachlan Kwok^1^, Sk Al Zaheri Mahmud^1^, Dr. Karen Hakobyan^1^, Prof. Megan S. Lord^2^, Prof. Guangzhao Mao^1,3,*^

^1^School of Chemical Engineering, University of New South Wales, Sydney 2052, Australia

^2^Graduate School of Biomedical Engineering, University of New South Wales, Sydney 2052, Australia

^3^School of Engineering, Institute for Materials and Processes, The University of Edinburgh, Robert Stevenson Road, Edinburgh, EH9 3FB, UK

* Corresponding authors: Guangzhao Mao ([guangzhao.mao@ed.ac.uk](mailto:guangzhao.mao@ed.ac.uk)) and Joel Yong ([joel.yong@unsw.edu.au](mailto:joel.yong@unsw.edu.au))

Figure S1. a) Average hydrodynamic diameter distribution of AuNP-MSA (black line) and AuNP-WGAHRP (red line) as measured by nanoparticle tracking analysis; modal peak ± standard error labelled above vertical dashed lines. Dotted lines represent standard error of distribution, n=5; b) Hydrodynamic diameter of resuspended lyophilized AuNP-WGAHRP after freezing by liquid nitrogen (snap freeze, black lines) and at a freeze rate of -1°C/min (slow freeze, blue lines), measured by multi angle dynamic light scattering. Solid lines denote intensity size and dotted lines denote number size. Data are representative of three consecutive measurements.

Table S1. Peak values from Figure S1b. Data represent mean ± standard deviation, n=3.

|  | Hydrodynamic diameter (nm) | |
| --- | --- | --- |
| Freeze method | Intensity size | Number size |
| Snap freeze (liquid nitrogen) | 15.0 ± 4.0  67.8 ± 11.1  371.1 ± 5.5 | 12.3 ± 2.4 |
| Slow freeze (-1°C/min) | 2.0 ± 0.2  11.5 ± 1.8  44.8 ± 4.9 | 1.9 ± 0.1 |


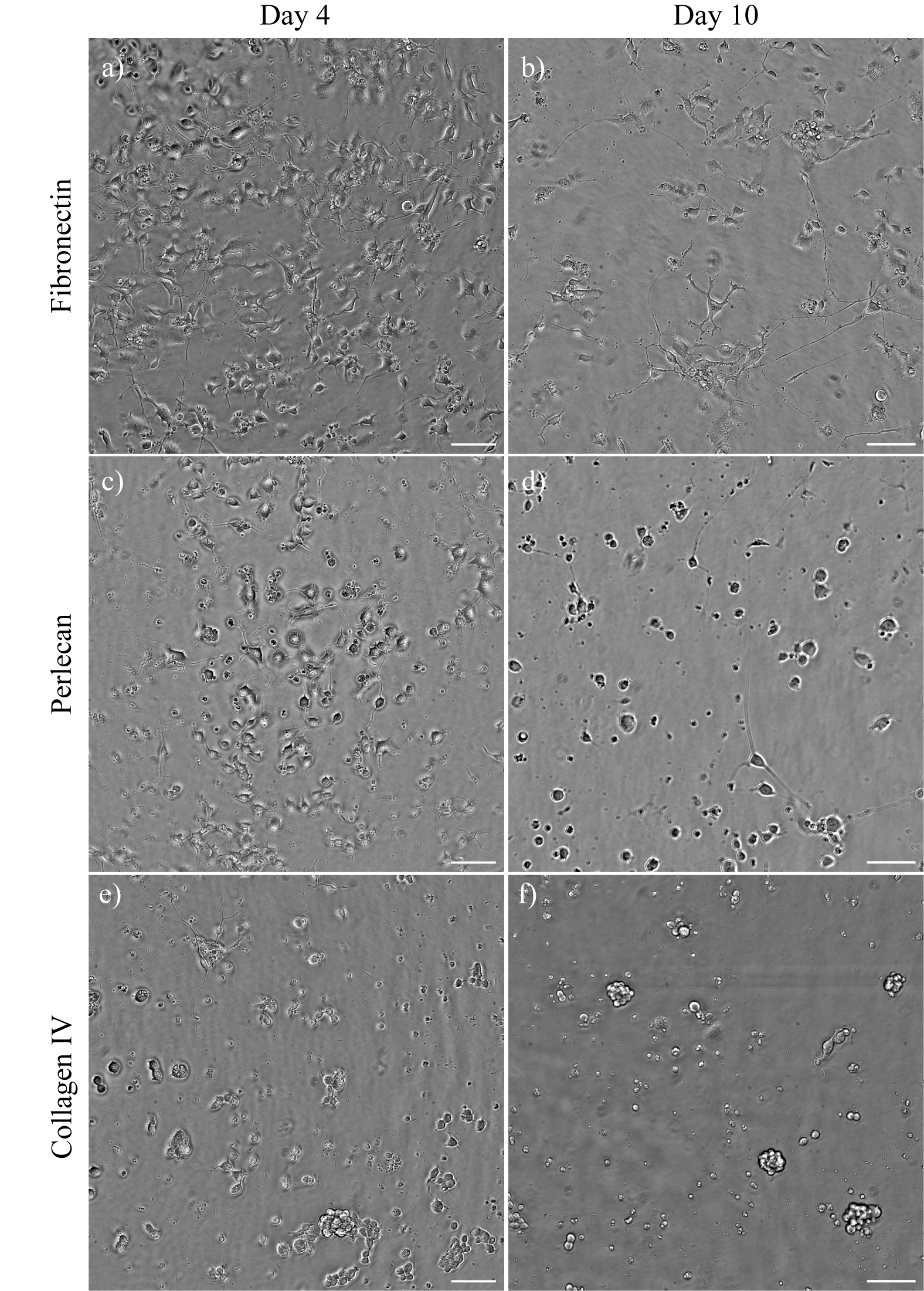


Figure S2. Phase contrast micrographs of NSC-34 cells plated on fibronectin (a, b) and perlecan (c,d) and collagen IV (e, f) for 4 days (a, c, e) and 10 days (b, d, f). Scale bars represent 100 µm.


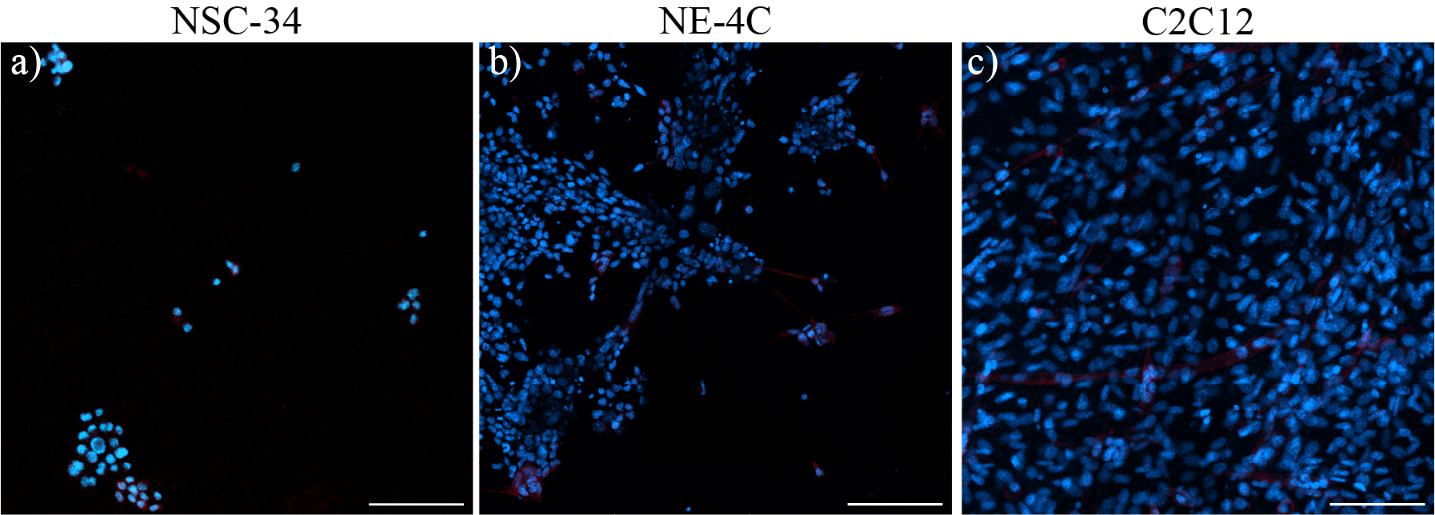


Figure S3. IgG isotype control staining of differentiated a) NSC-34, b) NE-4C and C2C12 cells, stained with goat anti-rabbit AlexaFluor 633 secondary antibodies (red) and Hoechst 33342 (blue)


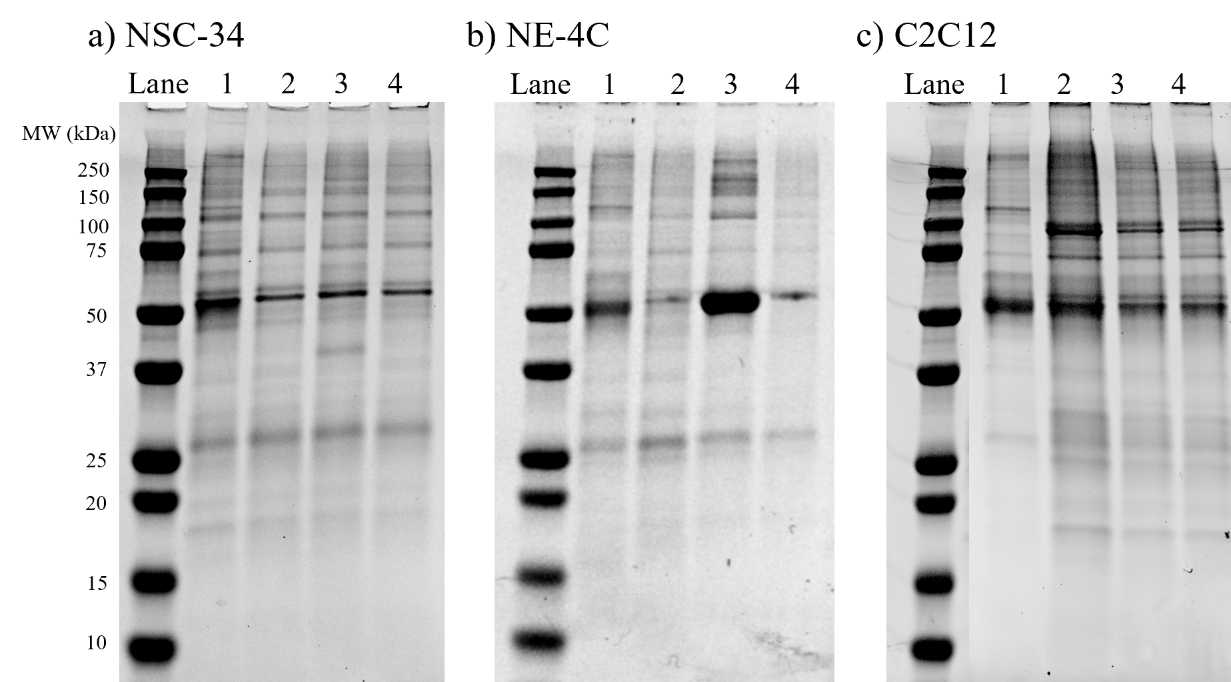


Figure S4. SDS-PAGE of purified glycoproteins from a) NSC-34 cells, b) NE-4C cells and c) C2C12 cells in the growth state (lanes 1) and differentiated states (lanes 2-4). Gels were stained with Coomassie.

Figure S5. a) ELLA comparing WGA-HRP (1.5 µg/ml) and AuNP-WGAHRP (0.145 µg/ml) binding to PVA or BSA blocked wells, growth state and differentiated state biological triplicate samples for three cell types. Bars and error bars represent mean and standard deviation respectively.

Figure S6. a) MST trace of FITC-Heparin alone, depicting the four phases of the MST response, I) steady state; II) T-jump; III) Thermophoresis; IV) inverse T-jump; b) Comparison of MST traces of FITC-Heparin alone or mixed with the highest concentration of WGA-HRP, AuNP-WGAHRP and AuNP-MSA; c-e) Raw fluorescence traces of FITC-heparin with WGAHRP, AuNP-WGAHRP and AuNP-MSA respectively. F_cold_ and F_hot_ are highlighted as grey regions.

MATERIALS AND REAGENTS

**WGA-HRP (Lectin from Triticum vulgaris, peroxidase conjugate) (Sigma-Aldrich Cat#L3892)**

CultureOne supplement (Thermo-Fisher Cat#A3320201)

DMEM with Glutamax and HEPES (Thermo-Fisher Cat no. 10564011)

Fetal bovine serum (Sigma-Aldrich Cat# F9423-500ml)

Hoescht 33342 (Thermo-Fisher Cat#H21492), prepared as a 10 mg mL^-1^ stock solution in water.

Neurobasal Plus medium (Thermo-Fisher Cat#A3582901)

Paraformaldehyde (Sigma-Aldrich Cat#P6148)

Penicillin-streptomycin (Sigma-Aldrich Cat#P4333-20ML)

Pierce Glycoprotein Isolation Kit (WGA) (Thermo-Fisher Cat#89805)

RIPA buffer without EDTA (Thermo-Fisher Cat# 89900)

TryplE reagent (Thermo-Fisher Cat#12604013)

CultureOne Supplement (Thermo-Fisher Cat# A3320201)

B27 Supplement (Thermo-Fisher Cat# 17504044)

N2 Supplement (Thermo-Fisher Cat#17502048)

ATTO-488 NHS ester (Sigma-Aldrich Cat#41051)

Pierce Protease inhibitor tables, EDTA-free (Thermo-Fisher Cat#A32965)

Ethylenediaminetetracetic acid disodium salt (EDTA) (UNIVAR, Cat#180-500G)

2,2'-azinobis[3-ethylbenzthiazoline-6-sulfonicacid] diammonium salt (ABTS) (Thermo-Fisher Cat#34026)

Laminin (Thermo-Fisher Cat#23017015)

**Antibodies:**

**Neurofilament 200 Antibody (Sigma Cat# N4142), used at 5 µg/ml**

**Rabbit IgG Isotype Antibody (Thermo-Fisher Cat# 02-6102), used at 15 µg/ml**

**α-bungarotoxin-AlexaFluor555 conjugate (Thermo-Fisher Cat#B35451), used at 6.25 µg/ml**

**Rabbit lectin – *Triticum vulgaris* Antibody (Sigma-Aldrich Cat#T4144-1VL), used at 5 µg/ml**

**Goat anti-rabbit IgG–AlexaFluor 633 (Thermo-Fisher Cat# A-21071), used at 10 µg/ml**

Lyophilisation method:

For snap freezing, 1 mL of AuNP-WGAHRP was frozen in a 15 ml Falcon tuber by liquid nitrogen. For slow freezing, 1 mL of AuNP-WGAHRP was frozen in Corning 1.2ml cryogenic vials (CLS430487) at a rate of -1°C/min by placing the cryogenic vials in a Corning CoolCell LX Cell freezing container and placing in a -80°C freezer overnight. For lyophilization, samples were placed in an Alpha 1-2 LDplus (Christ) freeze dryer for 24 hours. The freeze dryer was operated at -60°C and 0.001 mbar by a RV12 vacuum pump (Edwards).
